# Supplementary material for: NanoPipe—a web server for nanopore MinION sequencing data analysis
Source: Gigascience. 2019 Jan 24;8(2):giy169. doi: 10.1093/gigascience/giy169 (PMC6377397; doi:10.1093/gigascience/giy169)
Supplement: GIGA-D-18-00394_Original_Submission.pdf [file giy169_giga-d-18-00394_original_submission.pdf]

GigaScience

NanoPipe - a web server for nanopore MinION sequencing data analysis

--Manuscript Draft--

**Manuscript Number:**	GIGA-D-18-00394
**Full Title:**	NanoPipe - a web server for nanopore MinION sequencing data analysis
**Article Type:**	Technical Note
**Funding Information:**	Medizinische Fakultät, Westfälische Wilhelms-Universität Münster (n/a) — Dr. Wojciech Makalowski
**Abstract:**	Background<br><br>The fast-moving progress of the third generation long read sequencing technologies will soon bring the biological and medical sciences to a new era of research. Altogether the technique and experimental procedures are becoming more straightforward and available to biologists from diverse fields, even without any profound experience in DNA sequencing. Thus, the introduction of the MinIONTM device by Oxford Nanopore Technologies promises to “bring sequencing technology to the masses” and also allows quick and operative analysis in field studies. However, the convenience of this sequencing technology dramatically contrasts with the available analysis tools, which may significantly reduce enthusiasm of a “regular” user. To really bring the sequencing technology to every biologist, we need a set of user-friendly tools that can perform a powerful analysis in an automatic manner.<br><br>Findings<br><br>NanoPipe was developed in consideration of the specifics of the MinIONTM sequencing technologies, providing accordingly adjusted alignment parameters. The range of the target species/sequences for the alignment is not limited, and the descriptive usage page of NanoPipe helps a user to succeed with NanoPipe analysis. The results contain alignment statistics, consensus sequence, polymorphisms data, and visualization of the alignment. Several test cases are used to demonstrate efficiency of the tool.<br><br>Conclusions<br><br>Freely available NanoPipe software allows effortless and reliable analysis of MinIONTM sequencing data for experienced bioinformaticians, as well for wet-lab biologists with minimum bioinformatics knowledge. Moreover, for the latter group, we describe the basic algorithm of actions necessary for MinIONTM sequencing analysis from the first to last step.<br><br>Issue Section<br><br>TECHNICAL NOTE
**Corresponding Author:**	Wojciech Makalowski<br>Univeristy of Münster<br>Münster, GERMANY
**Corresponding Author Secondary Information:**	
**Corresponding Author's Institution:**	Univeristy of Münster
**Corresponding Author's Secondary Institution:**	
**First Author:**	Victoria Shabardina
**First Author Secondary Information:**	
**Order of Authors:**	Victoria Shabardina

Powered by Editorial Manager® and ProduXion Manager® from Aries Systems Corporation

	Tabea Kischka
	Felix Manske
	Norbert Grundmann
	Martin C. Frith
	Yutaka Suzuki
	Wojciech Makalowski
**Order of Authors Secondary Information:**	
**Additional Information:**	
**Question**	**Response**
Are you submitting this manuscript to a special series or article collection?	No
**Experimental design and statistics**<br><br>Full details of the experimental design and statistical methods used should be given in the Methods section, as detailed in our Minimum Standards Reporting Checklist. Information essential to interpreting the data presented should be made available in the figure legends.<br><br>Have you included all the information requested in your manuscript?	Yes
**Resources**<br><br>A description of all resources used, including antibodies, cell lines, animals and software tools, with enough information to allow them to be uniquely identified, should be included in the Methods section. Authors are strongly encouraged to cite Research Resource Identifiers (RRIDs) for antibodies, model organisms and tools, where possible.<br><br>Have you included the information requested as detailed in our Minimum Standards Reporting Checklist?	Yes
**Availability of data and materials**<br><br>All datasets and code on which the	Yes

Powered by Editorial Manager® and ProduXion Manager® from Aries Systems Corporation

conclusions of the paper rely must be either included in your submission or deposited in publicly available repositories (where available and ethically appropriate), referencing such data using a unique identifier in the references and in the "Availability of Data and Materials" section of your manuscript.<br><br>Have you have met the above requirement as detailed in our Minimum Standards Reporting Checklist?	

NanoPipe - a web server for nanopore MinION sequencing data analysis Click here to download Manuscript NanoPipe_manuscript.docx

NanoPipe - a web server for nanopore MinION sequencing data analysis

Victoria Shabardina[1], Tabea Kischka[1], Felix Manske[1], Norbert Grundmann[1], Martin C. Frith[2,3,4], Yutaka Suzuki[5], and Wojciech Makałowski[1]

[1] Institue of Bioinformatics, University of Muenster, Germany

[2] Artificial Intelligence Research Center, AIST, Japan

[3] Department of Computational Biology and Medical Sciences, The University of Tokyo, Japan

[4] AIST-Waseda University Computational Bio Big Data Open Innovation Laboratory, Japan

[5] Laboratory of Systems Genomics, Department of Computational Biology and Medical Sciences, The University of Tokyo

Corresponding author: Wojciech Makalowski, wojmak@uni-muenster.de

Abstract

Background: The fast-moving progress of the third generation long read sequencing technologies will soon bring the biological and medical sciences to a new era of research. Altogether the technique and experimental procedures are becoming more straightforward and available to biologists from diverse fields, even without any profound experience in DNA sequencing. Thus, the introduction of the MinION™ device by Oxford Nanopore Technologies promises to "bring sequencing technology to the masses" and also allows quick and operative analysis in field studies. However, the convenience of this sequencing technology dramatically contrasts with the available analysis tools, which may significantly reduce enthusiasm of a "regular" user. To really bring the sequencing technology to every biologist, we need a set of user-friendly tools that can perform a powerful analysis in an automatic manner.

Findings: NanoPipe was developed in consideration of the specifics of the MinION™ sequencing technologies, providing accordingly adjusted alignment parameters. The range of the target species/sequences for the alignment is not limited, and the descriptive usage page of NanoPipe helps a user to succeed with NanoPipe analysis. The results contain alignment statistics, consensus sequence, polymorphisms data, and visualization of the alignment. Several test cases are used to demonstrate efficiency of the tool.

Conclusions: Freely available NanoPipe software allows effortless and reliable analysis of MinION™ sequencing data for experienced bioinformaticians, as well for wet-lab biologists with minimum bioinformatics knowledge. Moreover, for the latter group, we describe the basic algorithm of actions necessary for MinION™ sequencing analysis from the first to last step.

Issue Section: TECHNICAL NOTE

Keywords: sequencing technologies, long reads sequencing, bioinformatics software, MinION, Oxford Nanopore

Background

Recent years have witnessed a DNA sequencing boom due to the constantly improving technologies and, consequently, the accessibility of sequencing to a large spectrum of customers including scientists and medical practitioners. Researchers in many fields, from metagenomics to plant physiology to medicine have been implementing sequencing experiments into their research. Oxford Nanopore Technologies (ONT) essentially accelerated this process by introducing the MinION™ sequencer, a portable device with minimum requirements for technical skills and bioinformatics knowledge. Thus, DNA sequencing experiments became feasible even in field studies, in small laboratories and soon will be available for medical applications in clinics [1].

The NCBI PubMed database includes 261 scientific articles containing "Oxford Nanopore" phrase published between 2009 and 2018 (by September 1, 2018), the majority of which were published in the last three years (see Fig. S1 in Supplementary). This is pointing, both, at the increased popularity of the ONT sequencers and at the considerable improvement of the technology and sequencing quality in the last three years. High throughput and long reads allow diverse applications of MinION™: virology [2], [3], plant pathology and agriculture [4], [5], tuberculosis studies [6], metagenomics and diet [7], veterinary research [8]; and as a portable platform: field biodiversity studies [9], detection of Ebola virus in patients on the spot [10], [11], sequencing in space [12]. Not to forget fundamental applications for long read sequencing studies, such as de novo genome assembly, improvement of existing genome assemblies and discovery of structural variants and long repeats [13]–[16]. ONT sequencing is favorable for microbiology research as small-sized

bacterial genomes can be covered in just one MinION™ read [17], [18], thus providing high resolution in genome architecture.

ONT supplies its users with the necessary software to perform base calling, i.e. converting of MinION™'s electrical signals into a sequence of nucleotides: the on-run MinKNOW and offline Albacore. Both applications utilize the complex, recurrent neural network (RNN) algorithm, which is recently very popular in computer science. It allows the software to learn from existing data and improve its performance. The output is a collection of FAST5 and/or FASTQ files containing the base-called sequences. These are the files that are used for any sequence analysis in bioinformatics, thus, the base-caller can be called a "gate" from MinION™ into data interpretation. Nevertheless, the range of ONT provided analysis tools is limited, and concerns only specific applications, excluding general processing, which is left to the user. For example, the EPI2ME software suite by ONT includes applications for barcode analysis, metagenomics and antimicrobial resistance analyses and some technical tests [19]. Several research groups have been recently focusing on the development of MinION™ specific bioinformatics tools [20]–[22], although most of these require considerable bioinformatics knowledge. These conditions impede benefits that MinION™ based DNA sequencing could bring to medical practitioners and researchers with less IT experience. To fill this gap, we developed NanoPipe, a web-driven automatic pipeline that can quickly and effortlessly process data produced by MinION™, as well provide necessary files for further bioinformatics analysis if required.

Methods

NanoPipe can be conceptually divided into four stages: 1) data uploading in FASTA or FASTQ formats, 2) alignment of MinION™ reads against the target sequences, 3) alignment analysis, and 4) results display (see Fig. 1). It was developed with no-IT-experienced users in mind, hence it provides a web-driven interface with the usage page describing the main

features of the tool. The start-of-analysis page is simple and intuitive. A user must, first, choose a target genome from a NanoPipe's list (see further) or upload their own target sequence. The next step is the essential part of the pipeline: mapping of sequencing reads to the target using the LAST sequence aligner (version 946). LAST accounts for the MinION™ specific sequencing errors, thus it generates highly reliable results. It can determine the rates of insertion, deletion and each kind of substitution in a type of data (e.g. MinION™ reads of AT-rich *Plasmodium* DNA) [23]. It then uses these rates to determine the most probable alignments. LAST also finds the most probable division of each read into one or more parts together with the most probable alignment of each part with the last-split function [24], [25], i.e. if LAST finds a better scoring alternative alignment of the read where it is being split into parts and mapped to different regions of the target, such alignment is submitted to the results. This is a principled way to handle complex DNA rearrangements, gene fusions in RNA, chimeric host/viral sequences, etc. The tasks of detecting polymorphisms and distinguishing viral serotypes can be performed more precisely for MinION™ data when based on a LAST alignment, because the tool estimates the probability that each base is correctly aligned. The probability is low if there is an ambiguity, i.e. the base could align to more than one place. The default NanoPipe parameters for the LAST alignment are efficient for most cases, but can be easily adjusted by the user. We use last-train to find the optimal alignment parameters for MinION™ sequencing [23]. For advanced users, it is recommended, although not necessary, to acquaint with the settings of LAST and last-train [26].

After the alignment is completed, NanoPipe evaluates the nucleotide variation for each position, and based on this analysis generates consensus sequence and a list of possible single nucleotide polymorphisms (SNP). The minimum nucleotide count per position should be at least ten (i.e., at least ten reads), otherwise a gap will be assigned at the

position in the consensus sequence. The consensus sequence is calculated based on the majority rule, i.e. the nucleotide with the higher count at a particular position is assigned to the consensus. If the counts for any two nucleotides differ from each other by not more than by 20%, both nucleotides are included in the consensus with the use of the IUPAC nomenclature [27]. Statistical evaluation of nucleotide variation is presented in a separate table, and suggests a polymorphism candidate if an alternate nucleotide has coverage of at least 20% of all reads.

Figure 1. A schematic representation of the NanoPipe workflow.

To distinguish between artifacts and true polymorphisms, we have set three additional filters. First, SNP candidate should have read coverage of at least 30% from the maximum coverage in the respective contig, otherwise it is not listed in the polymorphisms table and not counted as a SNP. Second, the probability of SNP at the position is calculated. For that, the relative nucleotide frequency is multiplied by a custom weight factor: transversions are weighted by the factor of 1 and transitions – by the factor of 2. This is based on the assumption that transitions are two times more likely than transversions. This weighted probability for each SNP candidate is rescaled for convenience, so that the maximum value is 1. The probability is displayed for each nucleotide in the polymorphisms table, a SNP candidate is most likely to be the true SNP if its joint probability is 1. Third, the analysis is refined by an assessment of the alignment quality around a potential SNP by estimating a p-error. The p-error is calculated based on the formula used in the LAST methods to calculate the probability of the alignment for a single nucleotide [28]. In NanoPipe the p-error is estimated over a region of maximum 10 nucleotides before and after the SNP position (excluding the SNP itself) and for all the read alignments at the region (Fig. 2). It is based on the LAST reliability score assigned to each base pair of the alignment. In addition,

polymorphism candidates for human and *Plasmodium* are linked to the public SNP databases.

$$p - error = \frac{\sum_{1}^{i} 10^{-\left(\frac{ASCII_{value}-33}{10}\right)}}{i}$$

Figure 2. Formula used in NanoPipe for the p-error calculations, where *i* is the total number of nucleotides around the SNP for all mapped reads, i.e. i = N(nucleotides around SNP)*N(reads mapped to the evaluated region). ASCII values are extracted from the LAST alignment and indicate the reliability of each base's alignment, see more information about LAST quality symbols at [28].

The results are supplemented with a number of useful pages, such as alignment of consensus sequences against target sequences, read length distribution and individual reads' alignment length distribution, and nucleotide plots showing distribution of nucleotides from all reads at each position of the consensus sequence. The latter is the interactive visual representation of the results and enables the user to monitor any nucleotide variations by eye. NanoPipe also provides the necessary files for browsing the alignment in the IGV genome browser [29], where each individual read alignment is displayed.

Findings

The experience of our working group in conducting MinION™ sequencing and analysis workshops [30] for medical doctors and wet-lab researchers revealed the general gaps in bioinformatics knowledge among these groups. While the experimental part of the workshop was easily performed by the participants, even if mastered for the first time, the processing of the sequenced data proved to be the most enduring and difficult part.

Therefore, keeping in mind the weakest spots of non-bioinformatician researchers, we will describe here some features of NanoPipe usage in details.

To start a new analysis, a query (sequencing reads) and a target (a genome or a region of interest) should be provided. The query (1D or $1D^2$) can be uploaded via the NanoPipe's web interface from the user's computer in one FASTA or FASTQ file, also in archived format. As the ONT-provided base callers output sequenced reads in multiple files, it is important to know how to merge them into one file, in an operating system of the user (Windows, Mac OS, Linux). As an alternative, NanoPipe can also handle multiple FASTA or FASTQ files when they are archived (zipped), for example, with "zip" command on Linux or WinZip tool on Windows. The maximum size of the query file should not exceed 3 GB. The target can be chosen from a drop-down menu or uploaded by the user in FASTA format. The list of NanoPipe precompiled targets includes reference genomes for human (hg38), RefSeq accession GCF_000001405.38; *Escherichia coli*, RefSeq accession GCF_000005845.2; *Caenorhabditis elegans*, RefSeq accession GCF_000002985.6; *Droshophila melanogaster*, RefSeq accession GCF_000001215.4; *Mus musculus*, RefSeq accession GCF_000001635.26; *Arabidopsis thaliana*, RefSeq accession GCF_000001735.4; *Plasmodium falciparum* strain 3D7, downloaded from plasmodb.org, version=2013-03-01; a representative genome for *Camponotus floridanus*, RefSeq accession GCF_003227725.1; and Dengue virus genome variants for serotyping (NC_001477.1, NC_001474.2, NC_001475.2 and NC_002640.1 for variant 1, variant 2, variant 3 and variant 4, respectively). Otherwise, DNA sequences of whole genomes, transcriptomes or genes for different organisms can be accessed via particular species databases or in the corresponding databases at NCBI [31]. The NanoPipe prepared targets include the precompiled, best fitting alignment parameters and substitution matrices (based on last-train calculations). The substitution matrix is an important part of any alignment and contains information about the mismatch cost

between any pair of nucleotides. Any uploaded targets will be used with the NanoPipe default parameters that are suitable for most cases. To avoid "noise", i.e. mapping of too short reads, the user can set the read length limit, for example to 200 nucleotides (depends on the experiment type and purpose). A unique name can be assigned to a job and used later within one month to retrieve the results from the NanoPipe server. After one month the data will be deleted from the server.

Depending on the query and target size, a NanoPipe analysis can last from several minutes to several hours, the server's memory used for the calculations is limited to 16 GB. The user will receive the notification via email (if the email address was provided) when his/her job is completed. The summary of the completed analysis depicts the LAST parameters that were used and the mapping statistics, i.e. how many reads were mapped altogether and the reads distribution per chromosomes/scaffolds of the target. This table can be sorted in increasing/decreasing order. Mapping distribution statistics show how long the reads in the query were, and allow estimating whether the sequencing resulted in the expected read lengths. Thus, for whole genome sequencing by MinION™ a researcher can expect read lengths up to several thousand nucleotides; targeted sequencing results in read lengths corresponding to the target length; transcriptome sequencing or RNAseq experiments should provide reads with length typical to particular species' transcript lengths (around one-to-two thousand nucleotides for most organisms). Alignment distribution statistics inform about the quality of the alignment, i.e. whether the whole read or a part of it could be mapped to the target.

Alignment results are visualized in NanoPipe in several ways: ordinal line by line pairwise alignment, a BAM file and a graphical representation via nucleotide plots. BAM and indexed BAM (BAI) files can be easily downloaded from the results page and further used for an interactive genome browsers, for example, IGV. The target FASTA files are required by IGV,

as well, and can be downloaded together with BAM files. IGV is free software and can be accessed from [32]. Nucleotide plots represent the colored mapping scheme at each position of the sequence, each nucleotide marked with its specific color. This enables easy monitoring for gaps and possible nucleotide substitutions. Navigating along the plot is enabled via right and left shift, as well by entering a nucleotide's coordinate in the search field. For long target regions, nucleotide plots provide a zoom-out preview at the bottom of the page.

Each chromosome/region of the target is supported with an individual consensus which can be seen and downloaded in FASTA format. Positions that cannot be defined (not enough information in the input data) are designated as "N", gaps are designated as "-"; controversial positions are designated using IUPAC nomenclature.

The polymorphisms table lists SNP candidates and provides joint probabilities for each candidate (maximum = 1), as well raw counts for each nucleotide at the target position. If the data are available, the corresponding SNP IDs (identifiers) will be retrieved from the existing databases (currently available only for human [33] and *Plasmodium* [34]). The alignment quality around an SNP candidate is reported as p-error, the higher the p-error (maximum 1), the lower the alignment quality. Low alignment quality might indicate a region of sequencing errors around an individual SNP and, thus, signify the lower reliability of the candidate detection. However, a cluster of closely located SNPs within a distance of less than 10 nucleotides would have a similar effect. Therefore, the p-error is an additional parameter that might be taken into consideration by the user. Detailed analysis of the nucleotide plot or the alignment in the IGV viewer around questionable candidates may help in making a decision, including consideration of biological relevance.

To view the result pages for nucleotide plots, consensus, polymorphisms and alignment the user needs to choose a particular chromosome/region. This approach accelerates the data display and prevents the web browser from overloading.

Study cases

Plasmodium polymorphism detection

The targeted regions were first amplified using the standard PCR protocol [35]. The resulting amplicons were sequenced with the MinION™ using the ONT sequencing kit SDK-MAP005 or SQK-MAP006 for the library preparation and the flow cell version FLO-MAP003. The sequences were aligned against the *Plasmodium* genome (*P. falciparum* reference genome based on 3D7 strain). Exhaustive discussion of the ONT utility for *Plasmodium* SNP calling and the library preparation methods are presented elsewhere [35]. NanoPipe succeeded in mapping 99.9% of all query reads and detecting the expected mutations (see Fig. 3). The specific characteristic of the *Plasmodium* genome, multiple AT/T repeats, can be easily observed in the nucleotide plot. The full analysis of a 21 MB (FASTA format) query against the *P. falciparum* genome with NanoPipe took less than four minutes. This example demonstrates that the analysis of just 10157 MinION™ reads with a high AT content on NanoPipe results in reliable data. Detailed screen shots of the NanoPipe results for this study case can be found in the Supplementary material (Figures S2 – S10).

Figure 3. Sample case 1. A: The polymorphisms table displays the three SNP candidates: two of them (at positions 403625 and 404407) are expected mutations leading to K76T and A220S amino acid changes and, as a consequence, to altered resistance of the parasite to chloroquine, mefloquine and quinine drugs [35]. B: Nucleotide plot. The purple arrow points to the G>T

substitution at the position 404407 (GCC>TCC codon change). The orange arrow highlights an AT-rich region, the typical feature of the *P. falciparum* genome.

Targeted sequencing of EGFR transcript from human lung adenocarcinoma cell line H1975

The region of the human EGFR cDNA corresponding to exons 17-22 was amplified using the primers CTAAGATCCCGTCCATCGCC (forward) and ACATATGGGTGGCTGAGGGA (reverse). The library preparation was performed following the manufacturer's recommendation using the SQK-LSK108 kit from ONT and then sequencing was done with the MinION™ using flow cell version FLO-MIN107 R9. The raw 1D reads (900 MB, FASTA format) were uploaded to NanoPipe using the human reference genome as a target; the analysis took 135 minutes. 79.9% of all reads were mapped to the EGFR gene on chromosome 7. Most of the remaining reads (13.5%) were mapped to chromosome 11, to regions corresponding to the cortactin gene (NM_138565) that is overexpressed in different cancers [36], and the gene encoding subunit 2 of the splicing factor 3b protein complex (NM_006842). This gene might be differentially expressed in tumor tissues [37], nevertheless, there is no definite research about possible roles of this gene in cancers. It may be the case that the gene is overexpressed in cell line H1975 and, therefore, its transcript was sequenced as an abundant contaminant, similar to the cortactin transcript. The four mutations expected within this region in this cell line [38] were detected, see Fig. 4. This example demonstrates the suitability of NanoPipe for cancer sequencing analysis.

Figure 4. A. Polymorphisms results. The two expected nucleotide substitutions are silent: G>A (CAG>CAA = Gln) at position 55181370 and T>C (ACT>ACC = Thr) at position 55198724; two other substitutions at position 55181378 (C>T leading to the amino acid change T745M) and at position 55191822 (T>G leading to the amino acid change L813R) are responsible for the sensitivity to anticancer drugs, in particular to gefitinib and erlotinib [38]. B. Nucleotide plot.

The zoom-out scheme at the bottom depicts seven alignment picks, they represent seven sequenced exons of the transcript. The nucleotide plot is showing the first of these picks, pointed to by the orange arrows.

RNAseq analysis of the ant species *Camponotus maculatus*

Poly-A RNA was extracted using the standard procedures, the library was prepared using the SQK-PCS108 kit from ONT and then sequenced with MinION™ using FLO-MIN107 R9 flow cell version following the manufacturer's recommendations. The raw 1D reads in FASTA format were uploaded to NanoPipe, the reference genome of *Camponotus floridanus* was used as the target genome, as neither genome nor transcriptome of *C. maculatus* are available yet. The analysis of the 1.5 GB query ran for 3 hours. Out of 1814750 raw reads 1773747 (97.7 %) were mapped to the target, spanning 431 scaffolds out of 657, including 150 scaffolds with coverage of more than 1000 reads per scaffold. This result is consistent with the number of reads sequenced, and the fact that the target was the genome of a different species, thus, NanoPipe can be used for studying newly sequenced species.

Discussion

We have developed NanoPipe, a web-driven application that enables easy analysis of MinION™ sequencing data and is suitable for, both, experienced bioinformaticians and biologists with limited IT knowledge. NanoPipe provides users with the consensus sequence of the studied DNA and a list of putative single nucleotide polymorphisms. In this work, we used three different experimental datasets: targeted sequencing of the AT-rich genome of *P. falciparum*, targeted sequencing of EGFR human cDNA, and RNAseq of the ant *P. maculatus* that has so far no fully sequenced genome nor transcriptome. These three test cases represent different tasks that biologists from different fields of study could be interested in: sequencing and SNP detection in a repeat-rich genomes with low sequencing

yield (study case with *P. falciparum*); mutation detection in human cancer samples (study case with lung adenocarcinoma cell line H1975); RNAseq of a newly sequenced organism (study case with *C. maculatus*). NanoPipe proved to be a reliable tool for all these tasks. It detected all expected mutations for *Plasmodium* and the EGFR transcript region and succeeded in mapping more than 90% RNAseq reads from *C. maculatus* to the reference genome. We have tested NanoPipe during two workshops at the International Summer School for MinION™ sequencing in Bangkok (2017) and Manado (2018) with participants having no bioinformatics experience from the medical and biological research fields. NanoPipe proved to be efficient and understandable for these users. Nevertheless, our team also noted during these practical studies that the participants lacked some basic knowledge, necessary for conducting successful sequencing experiments. Therefore, we described here in detail the general scheme of working with long read sequencing data. The flexibility of NanoPipe allows researchers to study any sequence of interest (including cancer samples in human patients), as the list of targets is not limited. With this software, we hope to make MinION™ sequencing even more accessible for medical researchers and biologists without sophisticated IT resources and expertise. The next step for the NanoPipe project will include more sophisticated statistics for SNP detection and evaluation. Also, we are planning to cover the microbiology field and implement an option for metagenomics analysis.

Availability of data and source code

Project name: NanoPipe

Project home page: http://bioinformatics.uni-muenster.de/tools/nanopipe, github page: https://github.com/IOB-Muenster/nanopipe2. The NanoPipe package for local installation is available at the NanoPipe github page.

Operating system: Unix

Programming language: Javascript, Python, Perl

License: Apache License 2.0

Other: there are no limitations for web browser type. The local version can be installed on Unix operating system

For reviewers: The raw sequence data for the test runs can be accessed from the homepage http://bioinformatics.uni-muenster.de/share/NanoPipe_test_data/

Test data description:

1. Data set *P. falciparum* sequencing

   1.1 MinION targeted sequencing for *Plasmodium falciparum* sequencing.

   1.2 PfCRT and K-propeller genes were sequenced using long-read technologies within the experiments of testing MinION sequencer for SNP detection in *Plasmodium*.

   1.3 The sequencing was performed for the MinION™ sequencing workshop, 2017 in Bangkok, Thailand at the Mahidol University.

   1.4 Raw reads in a single FASTA file, archived using zgip command.

   1.5 *P. falciparum* culture, strain 3D7.

   1.6 Size: 21 MB, archived: 4,3 MB.

2. Data set for EGFR sequencing

   2.1 MinION targeted sequencing of the CDS for EGFR human gene, spanning exons 17-22.

   2.2 This data were generated to test MinION capacity for detecting SNP in human cancer DNA

   2.3 The sequencing was performed for the MinION™ sequencing workshop, 2017 in Bangkok, Thailand at the Mahidol University.

   2.4 Raw reads in a single FASTA file, archived using zgip command.

   2.5 Human lung adenocarcinoma cell line H1975

   2.6 Size: 900 MB, archived: 259 MB.

3. Data set for *Camponotus maculatus* sequencing

3.1 MinION RNAseq for ant *C. maculatus*

3.2 Poly-A RNA sequencing was performed for the given ant species at first time

3.3 The sequencing was performed in the Institute of Bioinformatics, Muenster, Germany; the library was prepared in the group of Juergen Gadau, University of Muenster.

3.4 Raw reads in a single FASTA file, archived using zgip command.

3.5 Larvae and adult individuals from the lab culture

3.6 Size: 1.5 GB, archived: 309 MB.

The recent job IDs for the test cases in the text can be viewed in NanoPipe: 153789033639638 (*P. falciparum*), 153789042448841 (EGFR, human), 153789615015896 (*C. maculatus*).

Supplementary materials.

Figure S1: Number of scientific publications that contain the phrase "Oxford Nanopore" in their abstracts.

Figures S2-S10: The results pages for the *P. falciparum* test case.

Declarations

List of abbreviations

ONT Oxford Nanopore Technologies

SNP Single Nucleotide Variation

IT Information Technology

ID Identifier

GB GigaByte

Ethics approval and consent to participate

Not applicable

Consent for publication

Not applicable

Competing interests

The authors declare that they have no competing interests

Funding

The work has been funded by Institute of Bioinformatics, University of Muenster and University Clinic Muenster (UKM)

Authors' contribution

VS validated the software, participated in the concept design and composed the manuscript draft; TK designed the software and participated in the code writing; FM wrote the code for polymorphism analysis and contributed to the manuscript writing; NG wrote the major part of the pipeline code and designed the software; MF participated in the improvement of the software, including LAST optimization, and contributed to the manuscript writing; YS contributed to the analysis and provided the access to the MinION™ sequencing summer school; WM designed the concept and supervised the work. All the authors read and approved the manuscript.

Acknowledgements

The targeted sequencing of *Plasmodium falciparum* (strain 3D7) genes PfCRT_1, PfCRT_2, K13-propeller and EGFR human transcript was done for the MinION™ sequencing workshop in Bangkok, Thailand 2017 at the Mahidol University. The RNAseq library for *P. maculatus* was

prepared by the group of Jürgen Gadau, Molecular Evolution and Sociobiology group, University of Münster.

References

[1] ONT, "Flongle." [Online]. Available: https://nanoporetech.com/products/flongle. [Accessed: 05-Sep-2018].

[2] T. A. Sasani, K. R. Cone, A. R. Quinlan, and N. C. Elde, "Long read sequencing reveals poxvirus evolution through rapid homogenization of gene arrays," *bioRxiv*, p. 245373, Jan. 2018.

[3] T. Karamitros, I. Harrison, R. Piorkowska, A. Katzourakis, G. Magiorkinis, and J. L. Mbisa, "De Novo Assembly of Human Herpes Virus Type 1 (HHV-1) Genome, Mining of Non-Canonical Structures and Detection of Novel Drug-Resistance Mutations Using Short- and Long-Read Next Generation Sequencing Technologies," *PLoS One*, vol. 11, no. 6, p. e0157600, Jun. 2016.

[4] A. Bronzato Badial, D. Sherman, A. Stone, A. Gopakumar, V. Wilson, W. Schneider, and J. King, "Nanopore Sequencing as a Surveillance Tool for Plant Pathogens in Plant and Insect Tissues," *Plant Dis.*, vol. 102, no. 8, pp. 1648–1652, Aug. 2018.

[5] M. B. Fleming, E. L. Patterson, P. A. Reeves, C. M. Richards, T. A. Gaines, and C. Walters, "Exploring the fate of mRNA in aging seeds: protection, destruction, or slow decay?," *J. Exp. Bot.*, vol. 69, no. 18, pp. 4309–4321, Aug. 2018.

[6] A. Bainomugisa, T. Duarte, E. Lavu, S. Pandey, C. Coulter, B. J. Marais, and L. M. Coin, "A complete high-quality MinION nanopore assembly of an extensively drug-resistant Mycobacterium tuberculosis Beijing lineage strain identifies novel variation in repetitive PE/PPE gene regions," *Microb. Genomics*, Jun. 2018.

[7] W. Pearman, A. N. H. Smith, G. Breckell, J. Dale, N. E. Freed, and O. K. Silander, "New tools for diet analyses: nanopore sequencing of metagenomic DNA from stomach contents to quantify diet in an invasive population of rats," *bioRxiv*, p. 363622, Jul. 2018.

[8] S. Theuns, B. Vanmechelen, Q. Bernaert, W. Deboutte, M. Vandenhole, L. Beller, J. Matthijnssens, P. Maes, and H. J. Nauwynck, "Nanopore sequencing as a revolutionary diagnostic tool for porcine viral enteric disease complexes identifies porcine kobuvirus as an important enteric virus," *Sci. Rep.*, vol. 8, no. 1, p. 9830, Dec. 2018.

[9] H. Krehenwinkel, A. Pomerantz, J. B. Henderson, S. R. Kennedy, J. Y. Lim, V. Swamy, J. D. Shoobridge,

N. H. Patel, R. G. Gillespie, and S. Prost, “Nanopore sequencing of long ribosomal DNA amplicons enables portable and simple biodiversity assessments with high phylogenetic resolution across broad taxonomic scale,” *bioRxiv*, p. 358572, Jun. 2018.

[10] J. Quick, N. J. Loman, S. Duraffour, J. T. Simpson, E. Severi, L. Cowley, J. A. Bore, R. Koundouno, G. Dudas, A. Mikhail, N. Ouédraogo, B. Afrough, A. Bah, J. H. J. Baum, B. Becker-Ziaja, J. P. Boettcher, M. Cabeza-Cabrerizo, Á. Camino-Sánchez, L. L. Carter, J. Doerrbecker, T. Enkirch, I. G.- Dorival, N. Hetzelt, J. Hinzmann, T. Holm, L. E. Kafetzopoulou, M. Koropogui, A. Kosgey, E. Kuisma, C. H. Logue, A. Mazzarelli, S. Meisel, M. Mertens, J. Michel, D. Ngabo, K. Nitzsche, E. Pallasch, L. V. Patrono, J. Portmann, J. G. Repits, N. Y. Rickett, A. Sachse, K. Singethan, I. Vitoriano, R. L. Yemanaberhan, E. G. Zekeng, T. Racine, A. Bello, A. A. Sall, O. Faye, O. Faye, N. Magassouba, C. V. Williams, V. Amburgey, L. Winona, E. Davis, J. Gerlach, F. Washington, V. Monteil, M. Jourdain, M. Bererd, A. Camara, H. Somlare, A. Camara, M. Gerard, G. Bado, B. Baillet, D. Delaune, K. Y. Nebie, A. Diarra, Y. Savane, R. B. Pallawo, G. J. Gutierrez, N. Milhano, I. Roger, C. J. Williams, F. Yattara, K. Lewandowski, J. Taylor, P. Rachwal, D. J. Turner, G. Pollakis, J. A. Hiscox, D. A. Matthews, M. K. O. Shea, A. M. Johnston, D. Wilson, E. Hutley, E. Smit, A. Di Caro, R. Wölfel, K. Stoecker, E. Fleischmann, M. Gabriel, S. A. Weller, L. Koivogui, B. Diallo, S. Keïta, A. Rambaut, P. Formenty, S. Günther, and M. W. Carroll, “Real-time, portable genome sequencing for Ebola surveillance,” *Nature*, vol. 530, no. 7589, pp. 228–232, Feb. 2016.

[11] P. Mbala Kingebeni, C.-J. Villabona-Arenas, N. Vidal, J. Likofata, J. Nsio-Mbeta, S. Makiala-Mandanda, D. Mukadi, P. Mukadi, C. Kumakamba, B. Djokolo, A. Ayouba, E. Delaporte, M. Peeters, J.-J. Muyembe Tamfum, and S. Ahuka Mundeke, “Rapid confirmation of the Zaire Ebola Virus in the outbreak of the Equateur province in the Democratic Republic of Congo: implications for public health interventions,” *Clin. Infect. Dis.*, Jun. 2018.

[12] S. L. Castro-Wallace, C. Y. Chiu, K. K. John, S. E. Stahl, K. H. Rubins, A. B. R. McIntyre, J. P. Dworkin, M. L. Lupisella, D. J. Smith, D. J. Botkin, T. A. Stephenson, S. Juul, D. J. Turner, F. Izquierdo, S. Federman, D. Stryke, S. Somasekar, N. Alexander, G. Yu, C. E. Mason, and A. S. Burton, “Nanopore DNA Sequencing and Genome Assembly on the International Space Station,” *Sci. Rep.*, vol. 7, no. 1, p. 18022, Dec. 2017.

[13] E. A. Solares, M. Chakraborty, D. E. Miller, S. Kalsow, K. Hall, A. G. Perera, J. J. Emerson, and R. S. Hawley, “Rapid Low-Cost Assembly of the Drosophila melanogaster Reference Genome Using Low-Coverage, Long-Read Sequencing.,” *G3 (Bethesda).*, p. g3.200162.2018, Jul. 2018.

[14] S. Deschamps, Y. Zhang, V. Llaca, L. Ye, G. May, and H. Lin, “A chromosome-scale assembly of the sorghum genome using nanopore sequencing and optical mapping,” *bioRxiv*, p. 327817, May 2018.

[15] B. Pradhan, T. Cajuso, R. Katainen, P. Sulo, T. Tanskanen, O. Kilpivaara, E. Pitkänen, L. A. Aaltonen, L. Kauppi, and K. Palin, “Detection of subclonal L1 transductions in colorectal cancer by long-distance inverse-PCR and Nanopore sequencing,” *Sci. Rep.*, vol. 7, no. 1, p. 14521, Dec. 2017.

[16] L. F. K. Kuderna, E. Lizano, E. Julia, J. Gomez-Garrido, A. Serres-Armero, M. Kuhlwilm, R. A. Alandes, M. Alvarez-Estape, T. Alioto, M. Gut, I. Gut, M. H. Schierup, O. Fornas, and T. Marques-Bonet, “Selective single molecule sequencing and assembly of a human Y chromosome of African origin,” *bioRxiv*, p. 342667, Jun. 2018.

[17] K. Chacon-Vargas, A. A. Chirino, M. M. Davis, S. A. Debler, W. R. Haimer, J. J. Wilbur, X. Mo, B. W. Worthing, E. G. Wainblat, S. Zhao, and J. G. Gibbons, “Genome Sequence of *Zymomonas mobilis* subsp. *mobilis* NRRL B-1960,” *Genome Announc.*, vol. 5, no. 30, Jul. 2017.

[18] A. C. Reis, K. Kroll, M. Gomila, B. A. Kolvenbach, P. F. X. Corvini, and O. C. Nunes, “Complete Genome Sequence of *Achromobacter denitrificans* PR1,” *Genome Announc.*, vol. 5, no. 31, Aug. 2017.

[19] Metrichor, “METRICHOR LTD.” [Online]. Available: https://metrichor.com/. [Accessed: 05-Sep-2018].

[20] N. J. Loman, J. Quick, and J. T. Simpson, “A complete bacterial genome assembled de novo using only nanopore sequencing data,” *Nat. Methods*, vol. 12, no. 8, pp. 733–735, Aug. 2015.

[21] A. Srivathsan, B. Baloğlu, W. Wang, W. X. Tan, D. Bertrand, A. H. Q. Ng, E. J. H. Boey, J. J. Y. Koh, N. Nagarajan, and R. Meier, “A MinION-based pipeline for fast and cost-effective DNA barcoding,” *bioRxiv*, p. 253625, Jan. 2018.

[22] R. M. Leggett, D. Heavens, M. Caccamo, M. D. Clark, and R. P. Davey, “NanoOK: multi-reference alignment analysis of nanopore sequencing data, quality and error profiles,” *Bioinformatics*, vol. 32, no. 1, p. btv540, Sep. 2015.

[23] M. Hamada, Y. Ono, K. Asai, M. C. Frith, and J. Hancock, “Training alignment parameters for arbitrary sequencers with LAST-TRAIN,” *Bioinformatics*, vol. 33, no. 6, p. btw742, Dec. 2016.

[24] M. C. Frith and R. Kawaguchi, “Split-alignment of genomes finds orthologies more accurately,” *Genome Biol.*, vol. 16, no. 1, p. 106, Dec. 2015.

[25] M. C. Frith and S. Khan, “A survey of localized sequence rearrangements in human DNA,” *Nucleic Acids Res.*, vol. 46, no. 4, pp. 1661–1673, Feb. 2018.

[26] Frith M., “LAST: genome-scale sequence comparison.” [Online]. Available: http://last.cbrc.jp/. [Accessed: 05-Sep-2018].

[27] IUPAC, “IUPAC Codes.” [Online]. Available: https://www.bioinformatics.org/sms/iupac.html. [Accessed: 05-Sep-2018].

[28] Frith M., “last-split.” [Online]. Available: http://last.cbrc.jp/doc/last-split.html. [Accessed: 05-Sep-2018].

[29] H. Thorvaldsdottir, J. T. Robinson, and J. P. Mesirov, “Integrative Genomics Viewer (IGV): high-performance genomics data visualization and exploration,” *Brief. Bioinform.*, vol. 14, no. 2, pp. 178–192, Mar. 2013.

[30] Y. Suzuki, “JSPS, Training Seminar for MinION.” [Online]. Available: http://www.cb.k.u-tokyo.ac.jp/suzukilab/minion/summary.html. [Accessed: 09-Oct-2018].

[31] NCBI, “National Center for Biotechnology Information.” [Online]. Available: https://www.ncbi.nlm.nih.gov/. [Accessed: 05-Sep-2018].

[32] IGV-page, “Home | Integrative Genomics Viewer.” [Online]. Available: http://software.broadinstitute.org/software/igv/. [Accessed: 05-Sep-2018].

[33] NCBI.dbSNP, “dbSNP.” [Online]. Available: https://www.ncbi.nlm.nih.gov/projects/SNP/. [Accessed: 10-Oct-2018].

[34] PlasmoDB, “PlasmoDB : The Plasmodium Genomics Resource.” [Online]. Available: http://plasmodb.org/plasmo/. [Accessed: 10-Oct-2018].

[35] L. R. Runtuwene, J. S. B. Tuda, A. E. Mongan, W. Makalowski, M. C. Frith, M. Imwong, S. Srisutham, L. A. Nguyen Thi, N. N. Tuan, Y. Eshita, R. Maeda, J. Yamagishi, and Y. Suzuki, “Nanopore sequencing of drug-resistance-associated genes in malaria parasites, Plasmodium falciparum,” *Sci. Rep.*, vol. 8, no. 1, p. 8286, Dec. 2018.

[36] C. Gattazzo, V. Martini, F. Frezzato, V. Trimarco, E. Tibaldi, M. Castelli, M. Facco, F. Zonta, A. M. Brunati, R. Zambello, G. Semenzato, and L. Trentin, “Cortactin, another player in the Lyn signaling pathway, is over-expressed and alternatively spliced in leukemic cells from patients with B-cell chronic lymphocytic leukemia.,” *Haematologica*, vol. 99, no. 6, pp. 1069–77, Jun. 2014.

[37] T. A. Lantto, I. Laakso, H. J. D. Dorman, T. Mauriala, R. Hiltunen, S. Kõks, and A. Raasmaja, “Cellular Stress and p53-Associated Apoptosis by Juniperus communis L. Berry Extract Treatment in the Human SH-SY5Y Neuroblastoma Cells.,” *Int. J. Mol. Sci.*, vol. 17, no. 7, Jul. 2016.

[38] A. Suzuki, M. Suzuki, J. Mizushima-Sugano, M. C. Frith, W. Makalowski, T. Kohno, S. Sugano, K. Tsuchihara, and Y. Suzuki, "Sequencing and phasing cancer mutations in lung cancers using a long-read portable sequencer.," *DNA Res.*, vol. 24, no. 6, pp. 585–596, Dec. 2017.

Figure1

Click here to download Figure Picture1.pdf

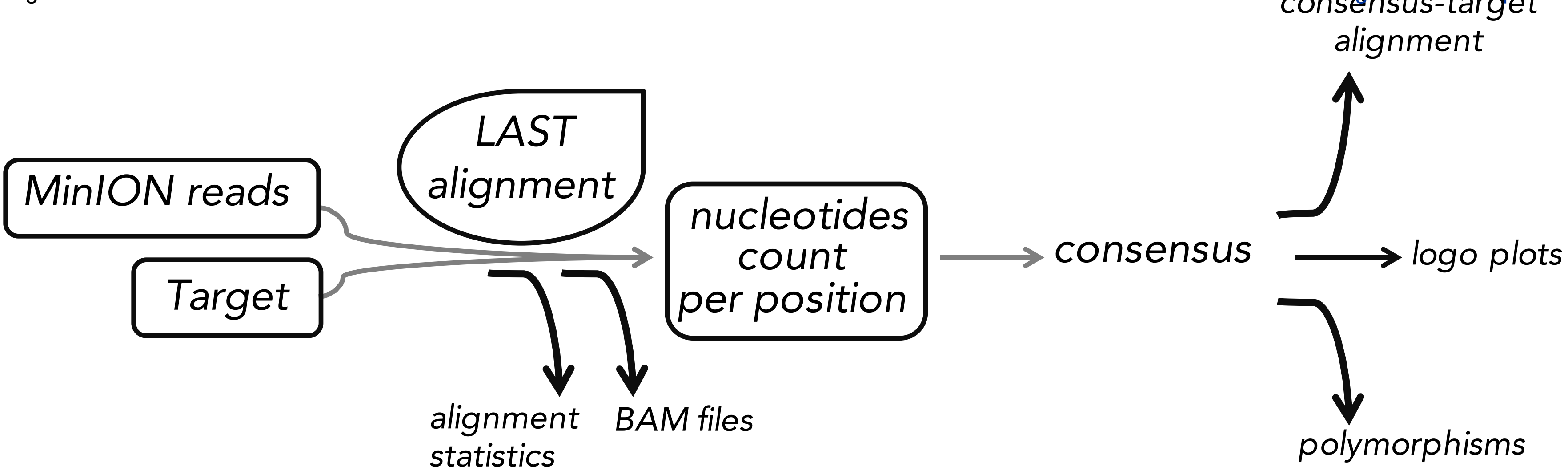

Figure3

Click here to download Figure Figure3.pdf

A

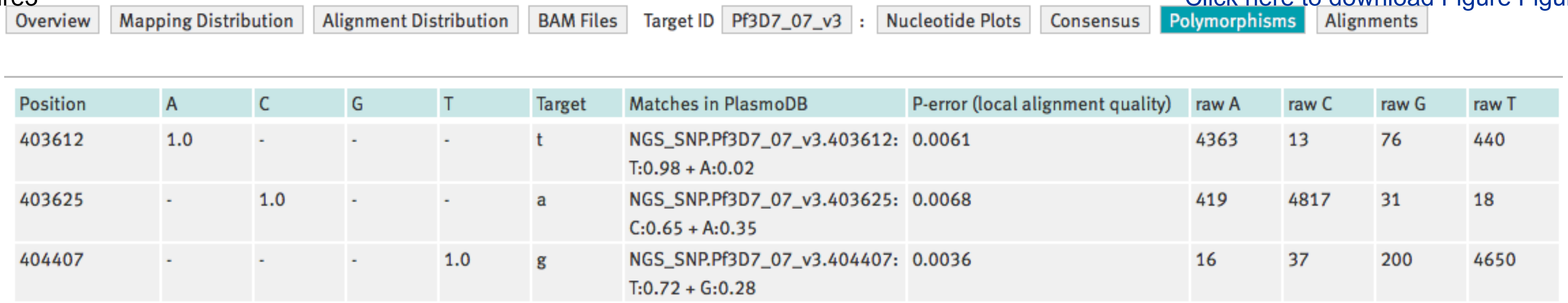

Position	A	C	G	T	Target	Matches in PlasmoDB	P-error (local alignment quality)	raw A	raw C	raw G	raw T
403612	1.0	-	-	-	t	NGS_SNP.Pf3D7_07_v3.403612: T:0.98 + A:0.02	0.0061	4363	13	76	440
403625	-	1.0	-	-	a	NGS_SNP.Pf3D7_07_v3.403625: C:0.65 + A:0.35	0.0068	419	4817	31	18
404407	-	-	-	1.0	g	NGS_SNP.Pf3D7_07_v3.404407: T:0.72 + G:0.28	0.0036	16	37	200	4650

B

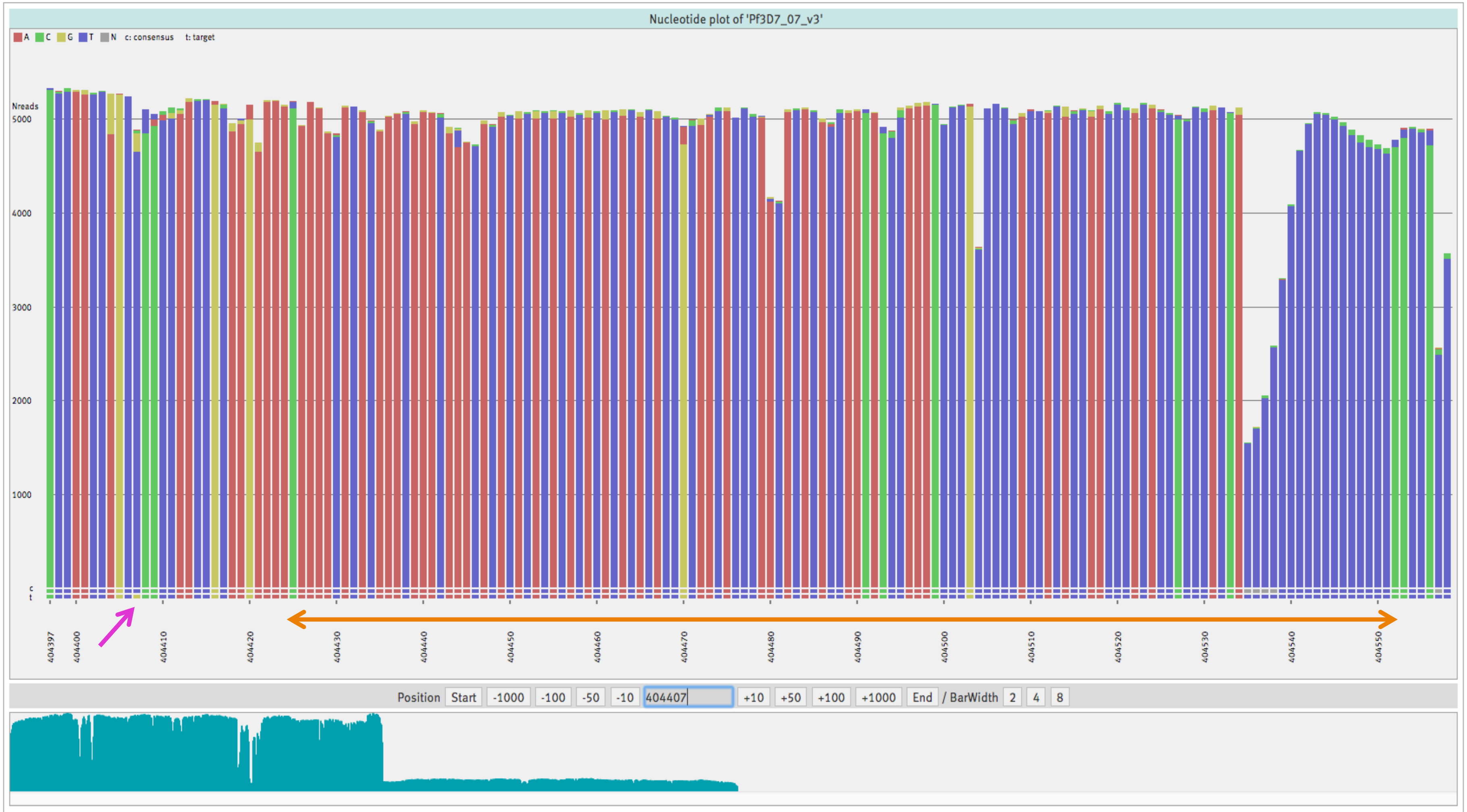

Figure4 Click here to download Figure Figure4.pdf

A

Position	A	C	G	T	Target	Matches in dbSNP	P-error (local alignment quality)	raw A	raw C	raw G	raw T
55173958	-	-	1.0	-	a		0.0027	104623	1304	38751	753
55181370	1.0	-	-	-	g	rs1050171: G/A+G/C	0.0008	114375	5449	75568	3831
55181378	-	-	-	1.0	c	rs121434569: C/T	0.0007	1423	65474	2980	128968
55191822	-	-	1.0	-	t	rs121434568: T/A+T/G	0.0013	19653	10419	75524	59396
55192839	-	-	1.0	-	a	rs376176117: A/T	0.0695	70026	211	18566	37
55198724	-	1.0	-	-	t	rs1140475: T/A+T/C	0.0563	1723	72518	896	49645

B

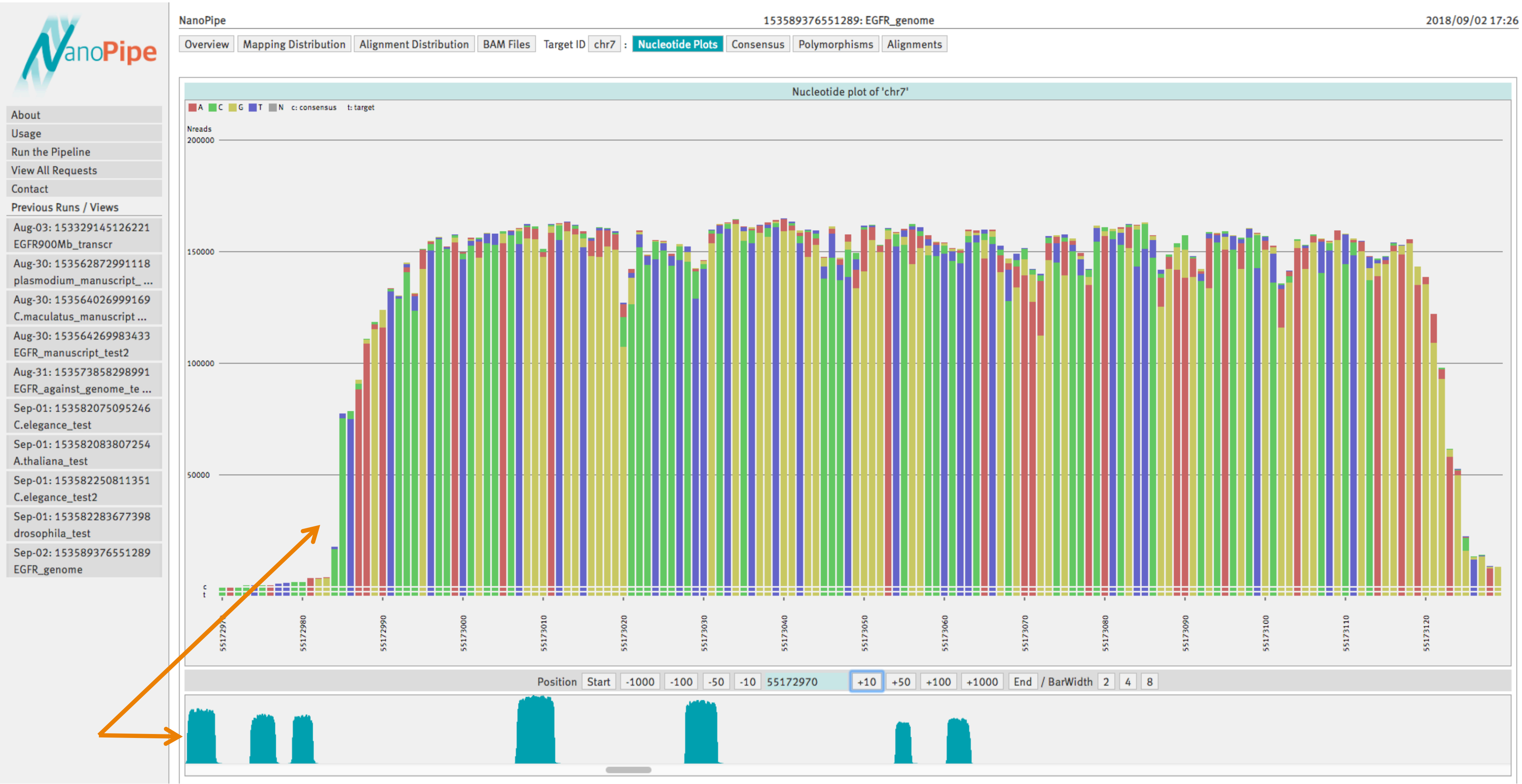

Click here to access/download
Supplementary Material
NanoPipe_Supplementary.pdf
