# Supplementary material for: NanoPipe—a web server for nanopore MinION sequencing data analysis
Source: Gigascience. 2019 Jan 24;8(2):giy169. doi: 10.1093/gigascience/giy169 (PMC6377397; doi:10.1093/gigascience/giy169)
Supplement: Supplemental File [file giy169_supplemental_file.pdf]

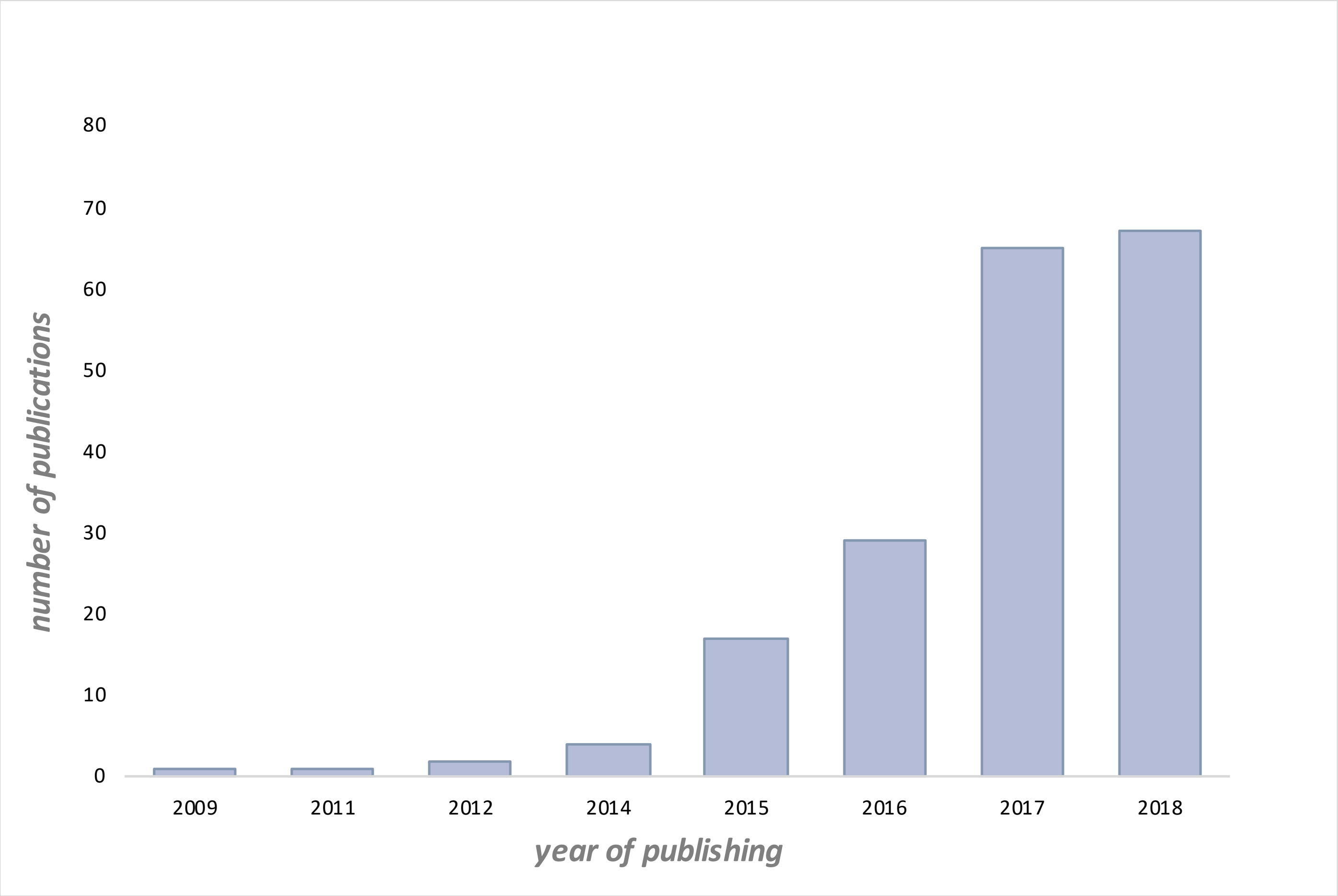

Figure S1. Number of scientific publications that contain the “Oxford Nanopore” phrase in their abstracts. Data were retrieved from the PubMED (https://www.ncbi.nlm.nih.gov/pubmed/) on 1.09.2018.

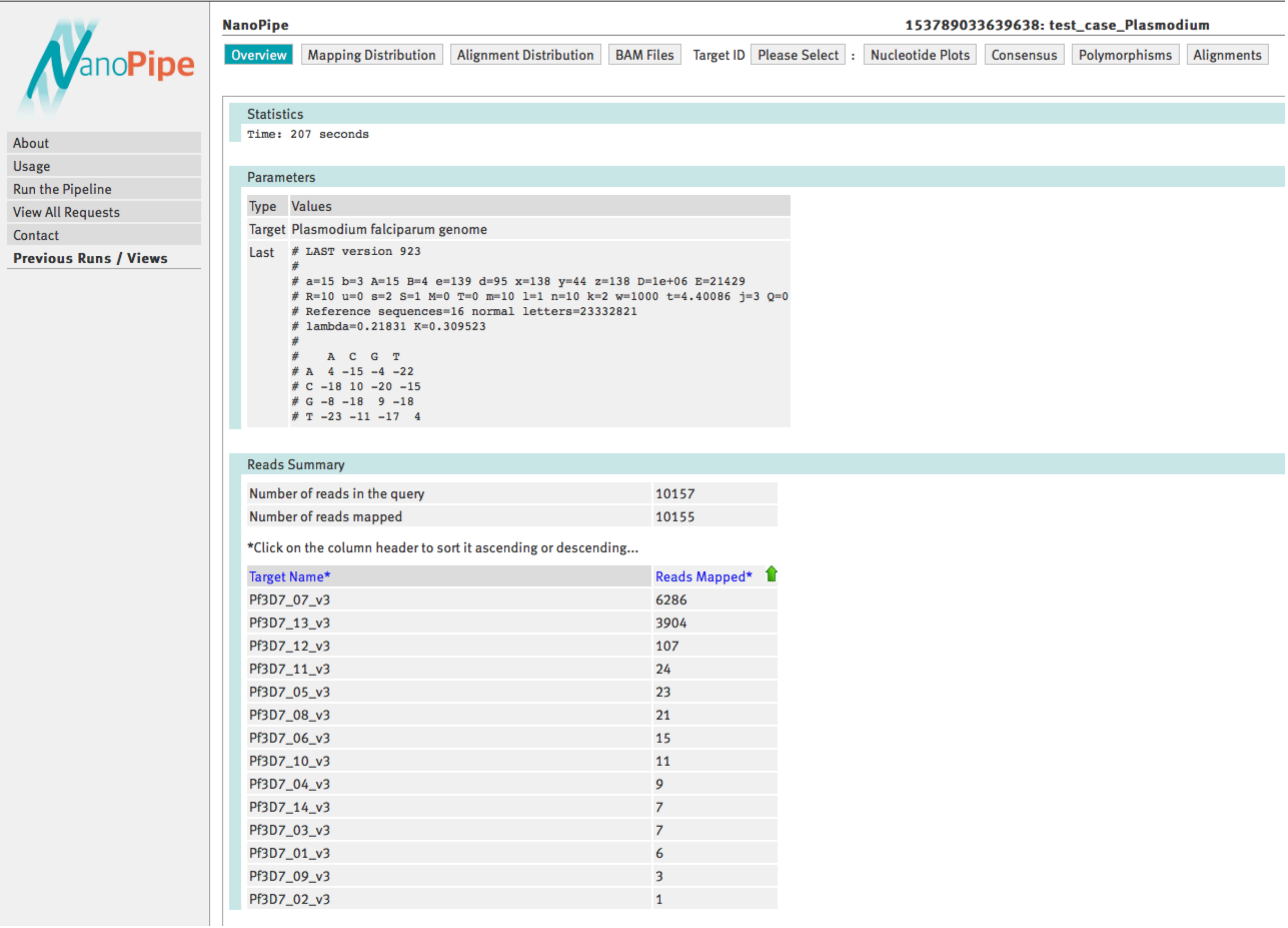

NanoPipe — 153789033639638: test_case_Plasmodium

Overview | Mapping Distribution | Alignment Distribution | BAM Files | Target ID | Please Select | : | Nucleotide Plots | Consensus | Polymorphisms | Alignments

About
Usage
Run the Pipeline
View All Requests
Contact
Previous Runs / Views

Statistics

```
Time: 207 seconds
```

Parameters

Type	Values
Target	Plasmodium falciparum genome
Last	# LAST version 923<br>#<br># a=15 b=3 A=15 B=4 e=139 d=95 x=138 y=44 z=138 D=1e+06 E=21429<br># R=10 u=0 s=2 S=1 M=0 T=0 m=10 l=1 n=10 k=2 w=1000 t=4.40086 j=3 Q=0<br># Reference sequences=16 normal letters=23332821<br># lambda=0.21831 K=0.309523<br>#<br># A C G T<br># A 4 -15 -4 -22<br># C -18 10 -20 -15<br># G -8 -18 9 -18<br># T -23 -11 -17 4

Reads Summary

Number of reads in the query	10157
Number of reads mapped	10155

*Click on the column header to sort it ascending or descending...

Target Name*	Reads Mapped*
Pf3D7_07_v3	6286
Pf3D7_13_v3	3904
Pf3D7_12_v3	107
Pf3D7_11_v3	24
Pf3D7_05_v3	23
Pf3D7_08_v3	21
Pf3D7_06_v3	15
Pf3D7_10_v3	11
Pf3D7_04_v3	9
Pf3D7_14_v3	7
Pf3D7_03_v3	7
Pf3D7_01_v3	6
Pf3D7_09_v3	3
Pf3D7_02_v3	1

Figure S2. Results page for *P. falciparum.* Overview.

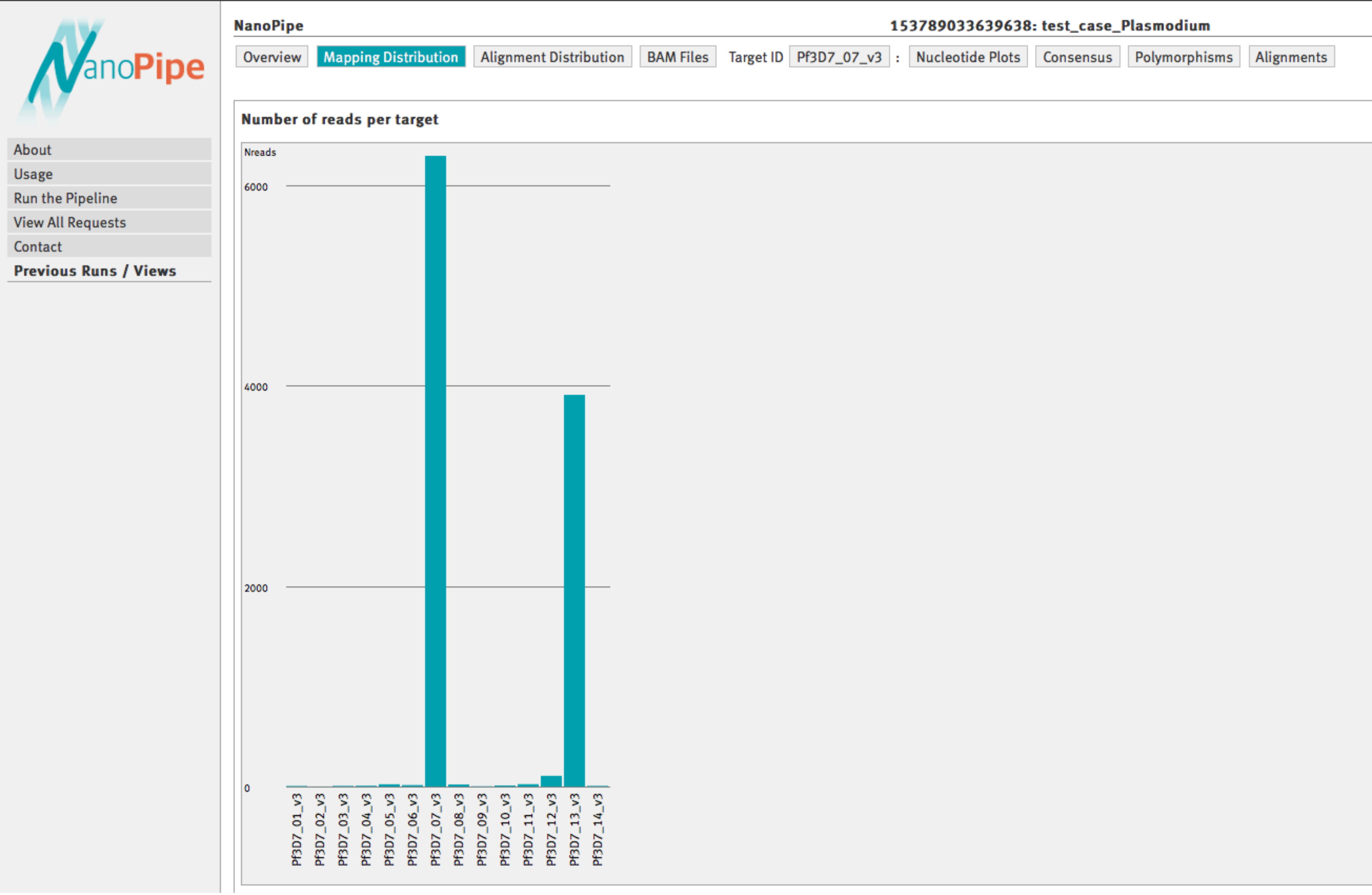

Figure S3. Results page for *P. falciparum.* Mapping distribution.

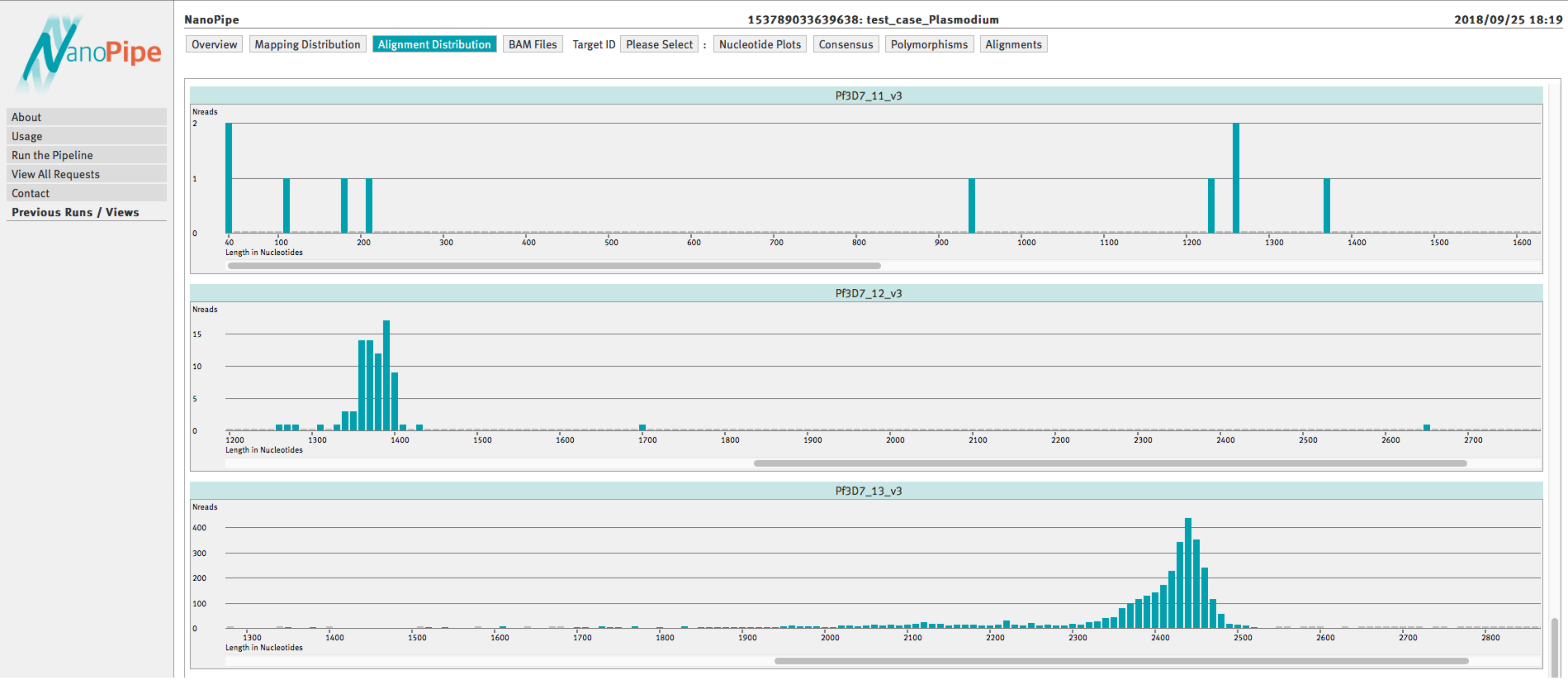

Figure S4. Results page for *P. falciparum.* Alignment distribution

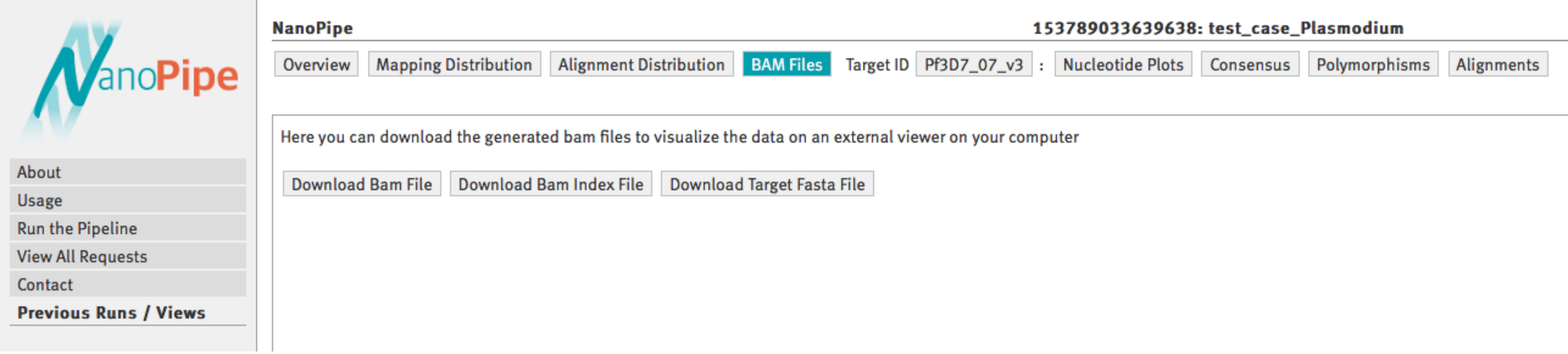

Figure S5. Results page for *P. falciparum.* BAM files.

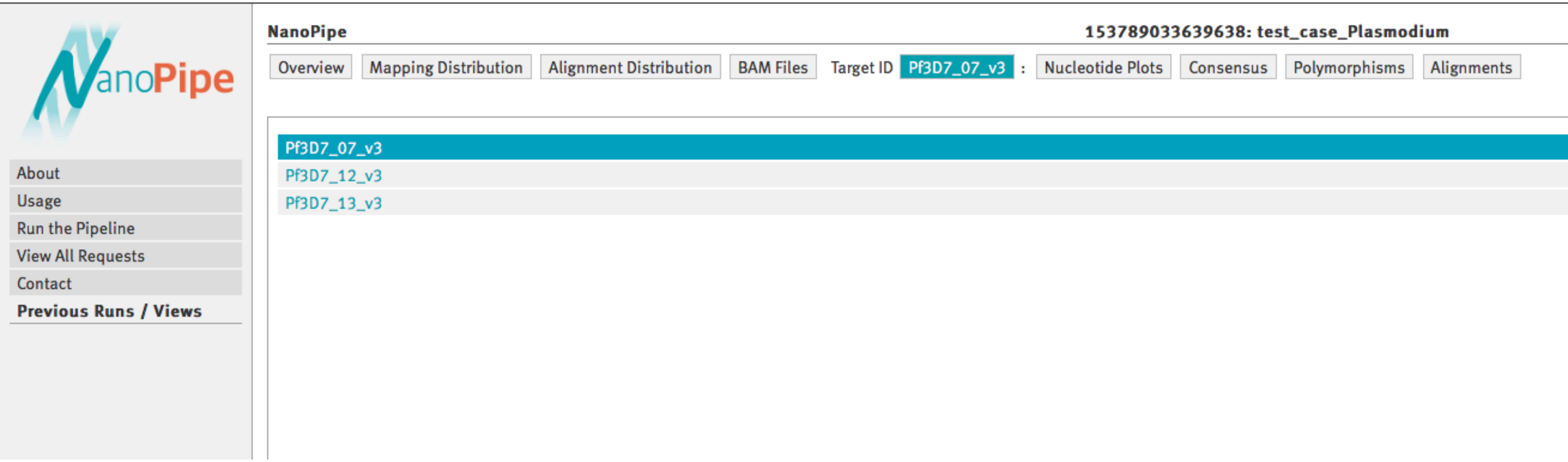

Figure S6. Results page for *P. falciparum*. Select target.

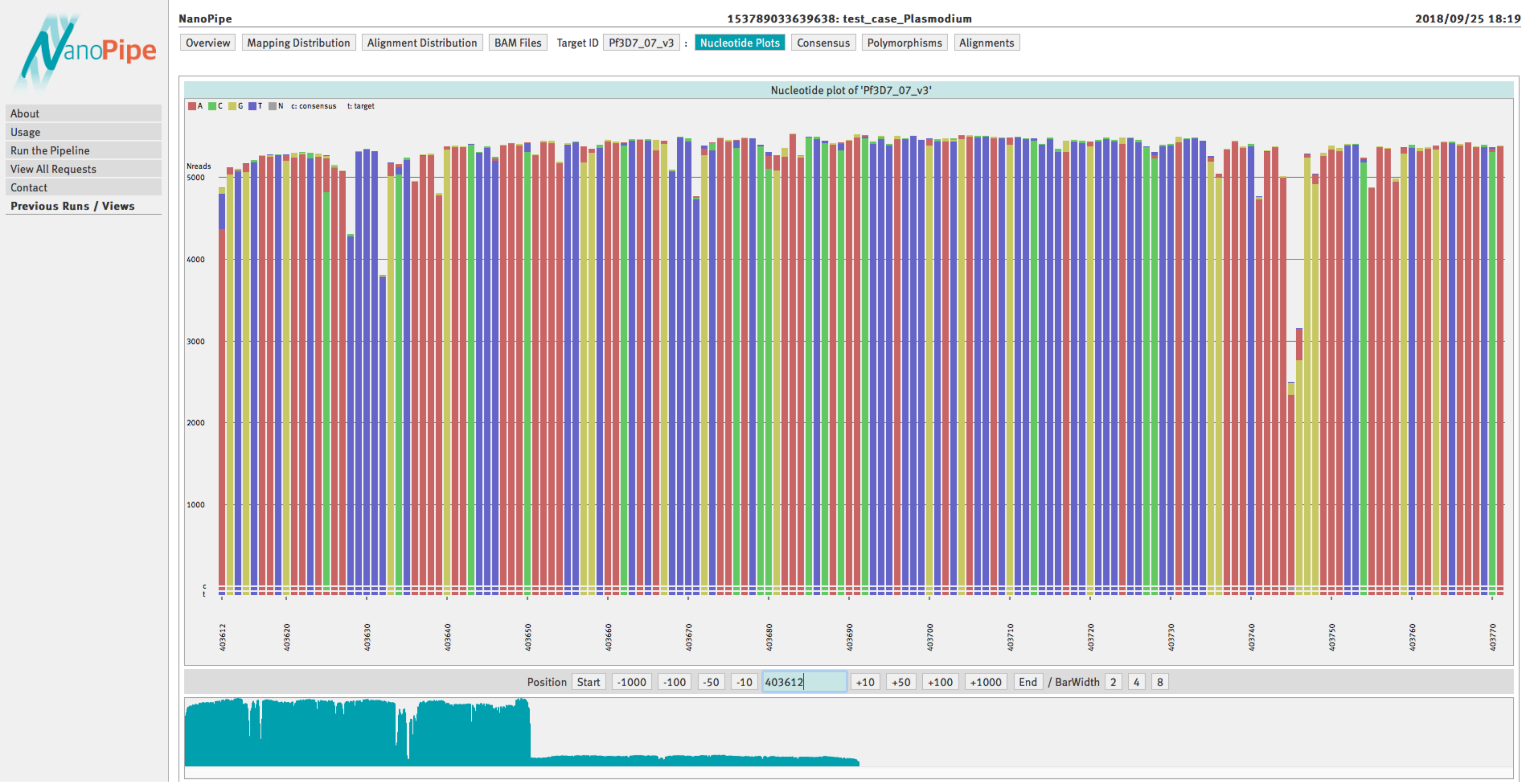

Figure S7. Results page for *P. falciparum.* Nucleotide plots.

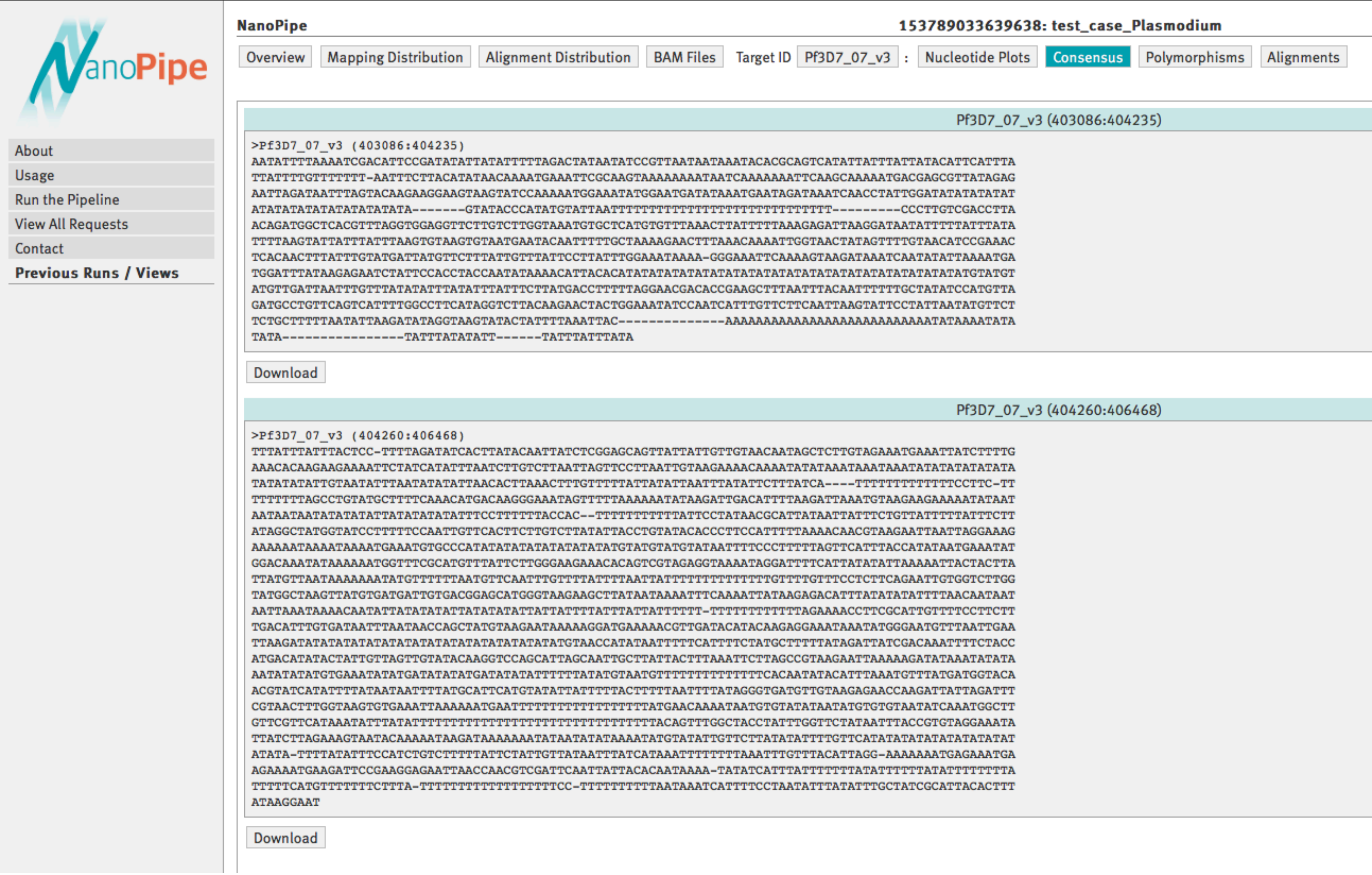

Figure S8. Results page for *P. falciparum.* Consensus sequence.

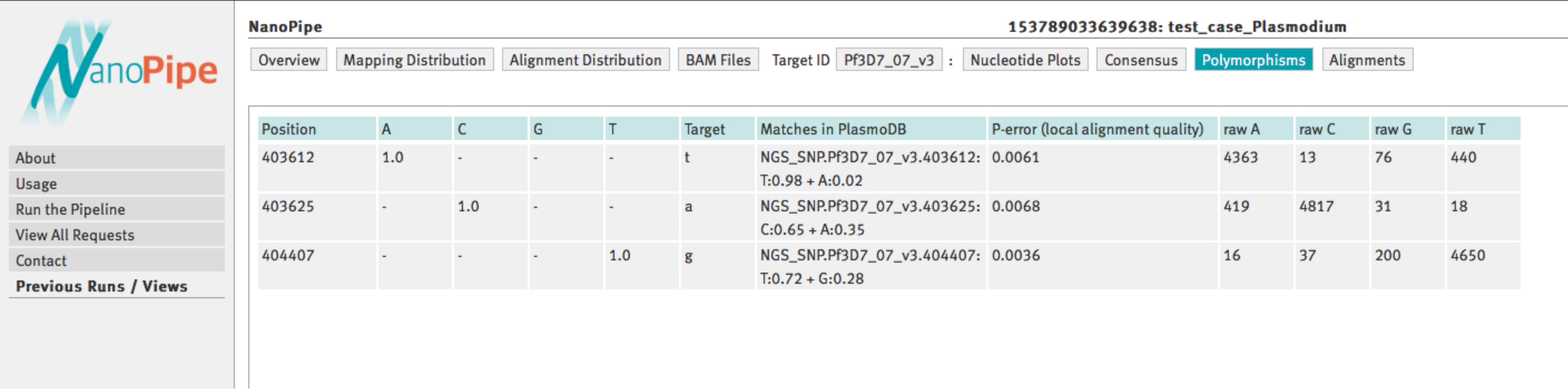

Position	A	C	G	T	Target	Matches in PlasmoDB	P-error (local alignment quality)	raw A	raw C	raw G	raw T
403612	1.0	-	-	-	t	NGS_SNP.Pf3D7_07_v3.403612: T:0.98 + A:0.02	0.0061	4363	13	76	440
403625	-	1.0	-	-	a	NGS_SNP.Pf3D7_07_v3.403625: C:0.65 + A:0.35	0.0068	419	4817	31	18
404407	-	-	-	1.0	g	NGS_SNP.Pf3D7_07_v3.404407: T:0.72 + G:0.28	0.0036	16	37	200	4650

Figure S9. Results page for *P. falciparum.* Polymorphisms.

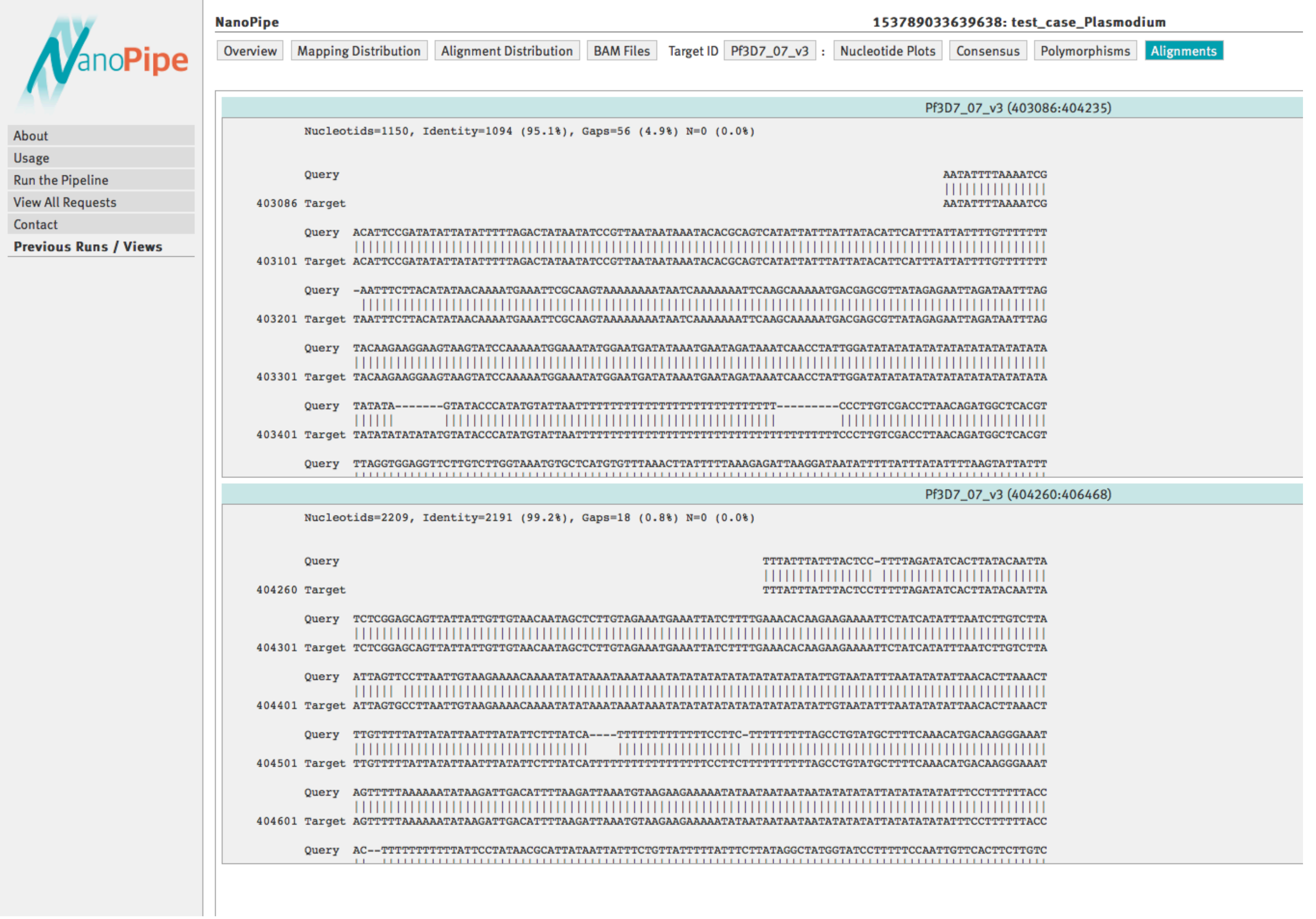

Figure S10. Results page for *P. falciparum.* Alignments.
