# Supplementary material for: NanoPipe—a web server for nanopore MinION sequencing data analysis
Source: Gigascience. 2019 Jan 24;8(2):giy169. doi: 10.1093/gigascience/giy169 (PMC6377397; doi:10.1093/gigascience/giy169)
Supplement: Response_to_Reviewer_Comments_Original_Submission.pdf [file giy169_response_to_reviewer_comments_original_submission.pdf]

Author's Response To Reviewer Comments

Close

Reply to reviewers' commentaries for Submission GIGA-D-18-00394

Dear Editor,
Thank you very much for processing our manuscript and giving the opportunity to work on its flaws and send the improved version. We also would like to thank the reviewers for the valuable criticism and attentive reading of the presented work. Their comments helped to improve our manuscript significantly. We hope that our replies and corresponding revisions of the manuscript are satisfactory and you will find the manuscript ready for publication at the GigaScience journal.

Sincerely,
Wojciech Makałowski

Reviewer 1.
Comment 1. A rather serious disagreement between the text and the web software is the set of reference genomes; the github archive appears to have no reference genomes. The manuscript claims 12 database and the 7 offered on the website don't fully overlap that set.

Reply: The manuscript and the NanoPipe menu, both, offer 9 pre-processed targets (page 8 of the manuscript): for human (hg38), RefSeq accession GCF_000001405.38; Escherichia coli, RefSeq accession GCF_000005845.2; Caenorhabditis elegans, RefSeq accession GCF_000002985.6; Droshophila melanogaster, RefSeq accession GCF_000001215.4; Mus musculus, RefSeq accession GCF_000001635.26; Arabidopsis thaliana, RefSeq accession GCF_000001735.4; Plasmodium falciparum strain 3D7, downloaded from plasmodb.org, version=2013-03-01; a representative genome for Camponotus floridanus, RefSeq accession GCF_003227725.1; and Dengue virus genome variants for serotyping (NC_001477.1, NC_001474.2, NC_001475.2 and NC_002640.1 for variant 1, variant 2, variant 3 and variant 4, respectively).
It is a good idea to keep all the information about targets in github repository: the list of targets and their source were added to the directory https://github.com/IOB-Muenster/nanopipe2/targets

Comment 2. I tried uploading a small set of E.coli reads and an E.coli reference (one of the manuscript-promised, not-on-server cases!) but I seem to be permanently parked behind two other jobs.
And Comment 3. The website has a "View Testcase" button -- that yields an error message screen.

Reply: We have tried the test case and the job run from the different computers, locations and web browsers and did not encounter any problems. Is it possible that the errors that the reviewer was experiencing had happened due to the local internet connection? What internet browser the reviewer used?

Comment 4. Similarly, the github package does not appear to contain a useful test case or any code to check the installation except running the package -- so if anything goes wrong it could be difficult for a novice to determine which of the long list of dependencies (11 Perl modules, 9 Python modules, 2 other tools)

Reply: The installation package deposited at github is aimed at users with some bioinformatics and coding knowledge and who would want to explore and modify the tool themselves. The novice users are offered to use the online version. Nevertheless, we thank the reviewer for this comment and agree that, indeed, it is helpful to know what has gone wrong, if anything has. We introduce the following improvements in the installation package:
1) The installation script has been written with the complementary explanations, please, see https://github.com/IOB-Muenster/nanopipe2/blob/master/install.sh and https://github.com/IOB-Muenster/nanopipe2/blob/master/install.txt
2) The check.sh script at the same directory (https://github.com/IOB-Muenster/nanopipe2) has been written for a user to be able to monitor if all the required packages are present on the user's computer.
3) A testcase has been deposited to check if the installation was successful. The install.txt file on the github repository includes description how to run the testcase. If the test script gives any errors, the user should double check the installation procedure and/or contact us via "Contact" option on the NanoPipe web page).

Comment 5. The authors might also consider distributing as a docker container and/or conda package with all dependencies covered.

Reply: Docker and conda are the good solutions for an installation packages, nevertheless we think that our install.sh script on github is covering well all the necessities. Besides, the accent of the tool is on its online application.

Comment 6. Streamlining the process of creating a new target database would be desirable -- the "install.txt" file gives a 5 step protocol -- two of which should be combined ("Create the target database" & "target.fasta". The lastdb step really should be wrapped in something that checks the new database information for consistency

Reply: The createtarget.sh script has been added to the https://github.com/IOB-Muenster/nanopipe2 github directory. It simplifies the target generation process. The explanation of usage can be found in the install.txt document.

Reviewer 2.
Part 1. Notes about the ONT technology
Thank you very much for the suggestions related to the introduction part; the facts from the Clive Brown's talk are, indeed, very exciting. Although we don't think that all the technical details should be included in our manuscript, since it is aimed at the broad audience, we have modified the text accordingly to give the general impression about the technology progressing. Please, see lines 59-61 and 75-77

Part2.
Comment 1. Introduction doesn't mention Metrichor, despite using TM in where MinION is used.

Reply: thank you for noting this, we filled that gap (line 82).

Comment 2. "for whole genome sequencing by MinION TM a researcher can expect read lengths up to several thousand nucleotides"
- longest read observed so far is 2.3 *million* nucleotides.

Reply: This information has been corrected (line 208).

Comment 3. MAP005/MAP006 kits and MAP003 flow cell in line 245 suggest a very old kit (~2-3 years old).This is unusual for a paper about to be published, but is consistent with one other GigaScience
paper that I've seen (?Sara Goodwin). I'd be interested to know how long this paper languished in pre-review doldrums until GigaScience accepted it for sending out to reviewers.
And: Line 270 for H1975 suggests SQK-LSK108/FLO-MIN107 R9; a bit more recent (but still old).

Reply: Indeed, the flow cells used for some experiments were not of the latest version. It depended on the collaboration we had had with the wet labs and the work progress. Nevertheless, the usage of not so recent flow cells in regard to the software development brings more robustness to the analysis, since the sequencing precision in this case is worse than with the newest equipment. We were able to show that the data processing with FLO-MAP003 and FLO-MIN107 flow cells provide with the reliable results. That ensures that the modern flow cells and sequencing kits will be not worse.

Comment 4. Line 292 -- what's the "standard procedures" for poly-A RNA extraction? Was this using poly-A bead selection? Why not strand switch sequencing with ONT adapters (available/recommended in ONT protocols from August 2017)?

Reply: We thank the reviewer for this comment: indeed, we happened not to be very precise in the description. The details about the RNA extraction has been added: please, refer to lines 297-298. We chose to use poly-A bead extraction method, because this procedure was optimized in our collaborators' lab to succeed with the RNA extraction from ant material. As the work with insect tissues is prone to difficulties, we did not want to change the established work flow in the lab that have expertise in working with ant species.

Comment 5. Given that this is a paper about *software*, I can't see any obvious reason why new samples were sequenced. It'd be nice to see this algorithm applied to recent large public human datasets (e.g. nanopore-wgs-consortium: ultra-long-read runs, or full-length RNA/cDNA
runs). Reads can be subset, as necessary, to cover particular genomic regions. This will help encourage people to use existing public datasets for their own software.

Reply: Following the suggestions of the reviewer and the editor we have added one more test case on the recently published direct RNA long read sequencing data of Vaccinia virus and its host. Please, see lines 308-319. The jobs IDs are 154401029652282 (mapped to the green monkey (host) genome) and 154400756783780 (mapped to the virus genome).

Comment 6. Text mentions that NanoPipe was used in Bangkok in 2017, but the github commits only go back to September 18 this year. Is there any reason why NanoPipe wasn't version controlled in 2017?

Reply: We were running NanoPipe through the series of tests and changes in 2017, that's why the github page was not created back then yet. The tool was at beta-version.

Comment 7. Note: IUPAC annotation for any of four nucleotides is 'N', not 'X' [https://github.com/IOB-Muenster/nanopipe2/blob/f2026e16b8942ec1cb60157b032a9c4bcbfebef7/modules/nanopipe2/calculate/analyze.pm#L341]
[https://www.bioinformatics.org/sms/iupac.html]

Reply: This, indeed, is different from IUPAC symbols. The issue is clarified in the manuscript, line 228, and in the usage explanations within the NanoPipe.

Close
