# Supplementary material for: NanoPipe—a web server for nanopore MinION sequencing data analysis
Source: Gigascience. 2019 Jan 24;8(2):giy169. doi: 10.1093/gigascience/giy169 (PMC6377397; doi:10.1093/gigascience/giy169)
Supplement: Reviewer_1_Report_original_submission -- Keith Robison — 11/27/2018 Reviewed [file giy169_reviewer_1_report_original_submission.pdf]

Reviewer Report

Title: NanoPipe - a web server for nanopore MinION sequencing data analysis

Version: Original Submission **Date:** 11/27/2018

Reviewer name: Keith Robison

Reviewer Comments to Author:

This application note describes a useful, web-oriented pipeline for several major nanopore data use cases.

Given the straightforward nature of the manuscript, I'm going to devote most (if not all) comments about the software itself in the hopes the authors find these useful

A rather serious disagreement between the text and the web software is the set of reference genomes; the github archive appears to have no reference genomes. The manuscript claims 12 database and the 7 offered on the website don't fully overlap that set.

I tried uploading a small set of E.coli reads and an E.coli reference (one of the manuscript-promised, not-on-server cases!) but I seem to be permanently parked behind two other jobs.

The website has a "View Testcase" button -- that yields an error message screen .

Similarly the github package does not appear to contain a useful test case or any code to check the installation except running the package -- so if anything goes wrong it could be difficult for a novice to determine which of the long list of dependencies (11 Perl modules, 9 Python modules, 2 other tools)

The authors might also consider distributing as a docker container and/or conda package with all dependencies covered.

Streamlining the process of creating a new target database would be desirable -- the "install.txt" file gives a 5 step protocol -- two of which should be combined (
"Create the target database" & "target.fasta". The lastdb step really should be wrapped in something that checks the new database information for consistency

Level of Interest

Please indicate how interesting you found the manuscript: Choose an item.

Quality of Written English

Please indicate the quality of language in the manuscript: Choose an item.

Declaration of Competing Interests

Please complete a declaration of competing interests, considering the following questions:

- Have you in the past five years received reimbursements, fees, funding, or salary from an organisation that may in any way gain or lose financially from the publication of this manuscript, either now or in the future?
- Do you hold any stocks or shares in an organisation that may in any way gain or lose financially from the publication of this manuscript, either now or in the future?
- Do you hold or are you currently applying for any patents relating to the content of the manuscript?
- Have you received reimbursements, fees, funding, or salary from an organization that holds or has applied for patents relating to the content of the manuscript?
- Do you have any other financial competing interests?
- Do you have any non-financial competing interests in relation to this paper?

If you can answer no to all of the above, write 'I declare that I have no competing interests' below. If your reply is yes to any, please give details below.

I declare that I have no competing interests

I agree to the open peer review policy of the journal. I understand that my name will be included on my report to the authors and, if the manuscript is accepted for publication, my named report including any attachments I upload will be posted on the website along with the authors' responses. I agree for my report to be made available under an Open Access Creative Commons CC-BY license (http://creativecommons.org/licenses/by/4.0/). I understand that any comments which I do not wish to be included in my named report can be included as confidential comments to the editors, which will not be published.

I agree to the open peer review policy of the journal
