# Supplementary material for: NanoPipe—a web server for nanopore MinION sequencing data analysis
Source: Gigascience. 2019 Jan 24;8(2):giy169. doi: 10.1093/gigascience/giy169 (PMC6377397; doi:10.1093/gigascience/giy169)
Supplement: Reviewer_2_Report_original_submission -- David Eccles — 11/30/2018 Reviewed [file giy169_reviewer_2_report_original_submission.pdf]

Reviewer Report

Title: NanoPipe - a web server for nanopore MinION sequencing data analysis

Version: Original Submission **Date:** 11/30/2018

Reviewer name: David Eccles

Reviewer Comments to Author:

This review is in two parts; the first part is notes about Clive Brown's presentation from the recent Nanopore Community meeting, which may be useful to add into the introduction. The second part includes some notes that I made when I first looked at this paper. Unfortunately I have not had sufficient time to combine these notes into something more cohesive, and give the paper another read-through. I didn't see any show-stopping issues with the manuscript, so have recommended that this paper be accepted for publication.

*** Part 1 of 2 ***

Here are some notes based on Clive Brown's slides at the Nanopore community meeting
(Wednesday 28th November, conference finished Thursday 29th November)
about flow cell performance as of December 2018
[https://vimeo.com/303444091]. Selected bits from these could be
included in the introduction as flavour text to talk about the current
state of nanopore sequencing:

* Unpolished homopolymer length accuracy is poor with current sequencing
pores (9.4.1), degrading from 100% for 3bp to close to 0% for
6bp. Upcoming R10 technology should improve that substantially (over
95% at 6bp).

* Consensus sequencing accuracy after polishing with medaka is about
q30-q35 with existing pores, q35-q43 with R10 pores.

* New flip-flop/CTC basecaller improves median accuracy to ~95%, can
be applied to existing nanopore fast5 reads. Gives close to an order
of magnitude improvement in consensus accuracy (from q28 to q36).

* 1DÂ² single molecule sequence accuracy should be improved to ~q20
with updated base-caller algorithms and unique pairing identifiers.

* Linear consensus sequencing (involving isothermal polymerisation)
targets over q30 for single molecules that are polymerised together

with replicative copies.

* Precision for detection of structural variants (via Minimap2 & Sniffles) is ~95%, recall is ~92% at 60x coverage, recall ~85% at 15x coverage.

* Cas9-based enrichment allows for 20-100x coverage of regions of interest on the $100 flongle flow cells.

* direct cDNA and direct RNA sequencing gives great quantitative accuracy on ERCC samples (rÂ² > 0.94). PCR cDNA Kit upgrades coming in december should allow for RNA inputs of 1ng and yields of 10M reads.

* MinION "revision D" flow cells (now available and in use by customers) have produced over 30 Gb from one flow cell (maximum customer yield). Cost $500-$900, depending on bulk purchase.

* Customer PromethION yield max 148 Gb from one flow cell, median 60 Gb (internally ONT is getting over 200 Gb). Rolling over existing improvements in MinION flow cells should push the yield over 300 Gb. Cost $975-$2000 per flow cell, depending on bulk purchase.

* Best flongle flow cell run 1.8 Gb, typical 0.5-1 Gb, cost $90 per flow cell.

*** Part 2 of 2 ***

Here are the bullet-point notes from my first run through the paper. I have not yet tried to use the software. In the interests of time, I'm giving this review as-is, without a review that includes running the software.

* Introduction doesn't mention Metrichor, despite using TM in where MinION is used.

* No other obvious errors in introduction, text seems to flow nicely.

* I like that it uses trained LAST; one of the great advantages of LAST over other alignment programs is that it allows for separate insertion and deletion probabilities, which is the case for the current MinION basecaller (i.e. deletions are more common than insertions).

Methods / line 203:

"for whole genome sequencing by MinION TM a researcher can expect
read lengths up to several thousand nucleotides"
- longest read observed so far is 2.3 *million* nucleotides.

MAP005/MAP006 kits and MAP003 flow cell in line 245 suggest a very old kit (~2-3 years old). This is unusual for a paper about to be published, but is consistent with one other GigaScience paper that I've seen (?Sara Goodwin). I'd be interested to know how long this paper languished in pre-review doldrums until GigaScience accepted it for sending out to reviewers.

Line 270 for H1975 suggests SQK-LSK108/FLO-MIN107 R9; a bit more recent (but still old).

Line 292 -- what's the "standard procedures" for poly-A RNA extraction? Was this using poly-A bead selection? Why not strand switch sequencing with ONT adapters (available/recommended in ONT protocols from August 2017)?

Given that this is a paper about *software*, I can't see any obvious reason why new samples were sequenced. It'd be nice to see this algorithm applied to recent large public human datasets (e.g. nanopore-wgs-consortium: ultra-long-read runs, or full-length RNA/cDNA runs). Reads can be subset, as necessary, to cover particular genomic regions. This will help encourage people to use existing public datasets for their own software.

** Software **

* Usage / documentation for the web service is a nice touch; thanks for adding this in.

* Text mentions that NanoPipe was used in Bangkok in 2017, but the github commits only go back to September 18 this year. Is there any reason why NanoPipe wasn't version controlled in 2017?

* Note: IUPAC annotation for any of four nucleotides is 'N', not 'X'
[https://github.com/IOB-Muenster/nanopipe2/blob/f2026e16b8942ec1cb60157b032a9c4bcbfebef7/modules/nanopipe2/calculate/analyze.pm#L341]
[https://www.bioinformatics.org/sms/iupac.html]

* It's good to see that the code is commented in places, with no obvious points where code has been commented out for testing / debugging; most recent code has summary information at the start of code functions, with additional explanation within the functions, where necessary.

Level of Interest

Please indicate how interesting you found the manuscript: Choose an item.

Quality of Written English

Please indicate the quality of language in the manuscript: Choose an item.

Declaration of Competing Interests

Please complete a declaration of competing interests, considering the following questions:

- Have you in the past five years received reimbursements, fees, funding, or salary from an organisation that may in any way gain or lose financially from the publication of this manuscript, either now or in the future?
- Do you hold any stocks or shares in an organisation that may in any way gain or lose financially from the publication of this manuscript, either now or in the future?
- Do you hold or are you currently applying for any patents relating to the content of the manuscript?
- Have you received reimbursements, fees, funding, or salary from an organization that holds or has applied for patents relating to the content of the manuscript?
- Do you have any other financial competing interests?
- Do you have any non-financial competing interests in relation to this paper?

If you can answer no to all of the above, write 'I declare that I have no competing interests' below. If your reply is yes to any, please give details below.

I have obtained complimentary and free replacement flow cells from Oxford Nanopore Technologies for my involvement in the ONT community, and accommodation / travel reimbursements for attendance at a PoreCamp in 2017.

I agree to the open peer review policy of the journal. I understand that my name will be included on my report to the authors and, if the manuscript is accepted for publication, my named report including any attachments I upload will be posted on the website along with the authors' responses. I agree for my report to be made available under an Open Access Creative Commons CC-BY license (http://creativecommons.org/licenses/by/4.0/). I understand that any comments which I do not wish to be included in my named report can be included as confidential comments to the editors, which will not be published.

I agree to the open peer review policy of the journal
